# Observation of a phonon bottleneck in copper-doped colloidal quantum dots

**DOI:** 10.1038/s41467-019-12558-y

**Published:** 2019-10-04

**Authors:** Lifeng Wang, Zongwei Chen, Guijie Liang, Yulu Li, Runchen Lai, Tao Ding, Kaifeng Wu

**Affiliations:** 1 State Key Laboratory of Molecular Reaction Dynamics and Dynamics Research Center for Energy and Environmental Materials, Dalian Institute of Chemical Physics, Chinese Academy of Sciences, 116023 Dalian, Liaoning China; 2 University of the Chinese Academy of Sciences, 100049 Beijing, China; 3 Hubei Key Laboratory of Low Dimensional Optoelectronic Materials and Devices, Hubei University of Arts and Science, 441053 Xiangyang, Hubei China

**Keywords:** Optical spectroscopy, Solar energy, Electronic properties and materials, Quantum dots

## Abstract

Hot electrons can dramatically improve the efficiency of solar cells and sensitize energetically-demanding photochemical reactions. Efficient hot electron devices have been hindered by sub-picosecond intraband cooling of hot electrons in typical semiconductors via electron-phonon scattering. Semiconductor quantum dots were predicted to exhibit a “phonon bottleneck” for hot electron relaxation as their quantum-confined electrons would couple very inefficiently to phonons. However, typical cadmium selenide dots still exhibit sub-picosecond hot electron cooling, bypassing the phonon bottleneck possibly via an Auger-like process whereby the excessive energy of the hot electron is transferred to the hole. Here we demonstrate this cooling mechanism can be suppressed in copper-doped cadmium selenide colloidal quantum dots due to femtosecond hole capturing by copper-dopants. As a result, we observe a lifetime of ~8.6 picosecond for 1*P*_*e*_ hot electrons which is more than 30-fold longer than that in same-sized, undoped dots (~0.25 picosecond).

## Abstract (web summary)

Weak electron-phonon scattering that can enable long-lived hot electrons in semiconductors is of interest in hot carrier solar cells. Here, the authors report copper-doped colloidal cadmium-selenide quantum dots with hot electron lifetime extended by more than 30-fold compared to undoped dots.

## Introduction

In conventional solar cells, the excessive energy of carriers above the band gap of a semiconductor is lost to heat due to rapid cooling (thermalization) of hot carriers via emission of phonons. This is a major reason for the well-known Shockley–Queisser limit for solar cell efficiency^1^. Hot carrier extraction to selective contacts has been envisioned as a promising means to overcome this limit, which has the potential to boost power conversion efficiency of solar cells to as high as 66%^2,3^. In addition, hot carriers can be utilized to improve the efficiency of photocatalysis or sensitize some very energetically demanding photochemical reactions. The challenge in efficient hot carrier extraction lies in that intraband cooling of hot carriers usually occurs on a sub-ps timescale.

**Fig. 1:**
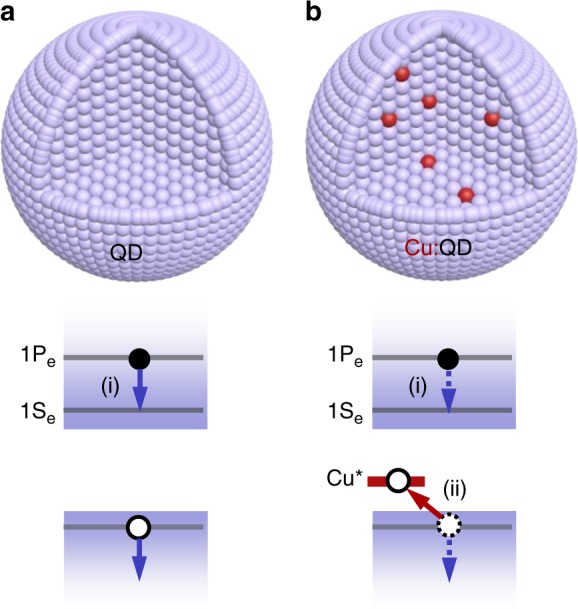
Suppression of hot electron cooling in Cu-doped QDs. **a** In typical QDs, hot electrons relax on a sub-ps timescale through an Auger-like process whereby the electron relaxation energy is used to excite the hole in the valence band, denoted as process (i). **b** In Cu:QDs, ultrafast hole capturing (ii) by the intragap copper states (Cu*) decouples electrons and holes, thus suppressing Auger cooling of electrons and enabling a phonon bottleneck for hot electrons

It is thus essential to suppress the rate of hot carrier cooling. In most inorganic materials, including semiconductors and metals, hot carriers relax on a sub-ps timescale via scattering with phonons. According to some recent reports, lead halide perovskites might be an exception, as the coupling of carriers to longitudinal optical (LO) phonons could be screened by the highly dynamic structure of these “liquid-like” crystals via polaron formation^4–6^. However, many transient absorption measurements revealed that under weak excitation power densities the majority of hot carriers still relaxed within 1 ps^6–12^. Another class of materials that was predicted to show long-lived hot carriers is colloidal semiconductor nanocrystals or quantum dots (QDs)^13,14^. Due to a strong quantum confinement effect, energy levels in colloidal QDs are sparsely spaced with the inter-level energy gap substantially higher than the LO phonon energy^15–17^. As a result, carrier cooling through LO phonons should be very inefficient, which is called a phonon bottleneck^14^. Contrary to this prediction, ultrafast spectroscopic measurements showed sub-ps lifetime of hot electrons in various types of QDs^18–23^. For common II–VI group QDs (such as CdSe), this observation has been rationalized using an Auger-like process whereby the excessive energy of the hot electron is transferred to a hole^18,24^, as denoted by process (i) in Fig. 1a. Note that the hot hole can quickly relax via phonons due to much more closely spaced energy levels in the valance band of II–VI QDs^16^.

Realizing the detrimental role of the Auger-type cooling mechanism, the key to enabling a phonon bottleneck and to prolonging hot electron lifetime is to decouple the electron from the hole and thus to suppress energy transfer between them. Indeed, hot electron lifetime on the order of tens of ps was demonstrated in CdSe/ZnS/ZnSe/CdSe core/multi-shell QDs featuring electron-hole separation, which could be further prolonged by using capping ligands of low infrared absorbance^25,26^. The issue with these complicated core/shell structures is that carrier extraction from them can be very difficult which renders them unsuited for solar energy conversion applications.

In principle, efficient suppression of hot electron cooling via the phonon bottleneck can be realized in a much simpler system, copper-doped QDs (Cu:QDs). These QDs have been developed for decades and have been extensively studied for their peculiar light emission and photo-induced magnetism properties^27–33^. These properties are enabled by ultrafast capturing of the photogenerated hole into intragap states associated with the Cu dopants (Cu*), as denoted by process (ii) in Fig. 1b. If this hole capturing process is faster than hot electron relaxation^30^, long-lived hot electrons would be expected for Cu:QDs. Such observations, however, have not yet been reported in ultrafast spectroscopic studies on Cu:QDs in the literature. Gamelin et al. studied carrier recombination dynamics in *n*-type Cu-doped CdSe/CdS QDs and found an ultrafast (~260 ps), Auger-dominated negative trion recombination pathway involving two band edge electrons and a Cu-captured hole^34^, but no hot carrier relaxation dynamics was reported in this work. Slightly longer-lived hot electrons in Cu:CdSe QDs as compared to CdSe QDs (700 fs vs. 400 fs) were reported in the works by Ghosh et al.^35^ and by Patra et al.^36^, but such a marginal improvement in hot electron lifetime is not sufficiently impactful for hot electron devices. Thus, in order to realize long-lived hot electrons in Cu:QDs, it is essential to optimize the hole capturing process by Cu dopants and to directly measure the rate of this process and compare it with hot electron relaxation rate.

Here we show that, in Cu:CdSe QDs prepared by a recently-introduced, one-pot method^37^, hole capturing by Cu* occurs on a ≪390 fs timescale and hence competes favorably with the sub-ps Auger-type energy transfer. As a result, electrons are effectively decoupled from holes (Fig. 1b), allowing us to observe a phonon bottleneck for hot electron relaxation in Cu:QDs.

## Results

### Structural and optical characterizations of Cu-doped QDs

The Cu:CdSe QDs were synthesized using a published procedure;^37^ see Methods for details. A representative transmission electron microscope (TEM) image shows an average diameter of 3.3 ± 0.4 nm for these Cu:CdSe QDs (Supplementary Fig. 1). Elemental composition analysis, performed using inductively coupled plasma atomic emission spectroscopy (ICP-AES), showed a Cu to Cd ratio of 1.8:100, similar to previously reported values^37^. As a control sample, we also synthesized same-sized, undoped CdSe QDs using similar synthetic conditions but without adding Cu-precursors (Supplementary Fig. 1).

**Fig. 2:**
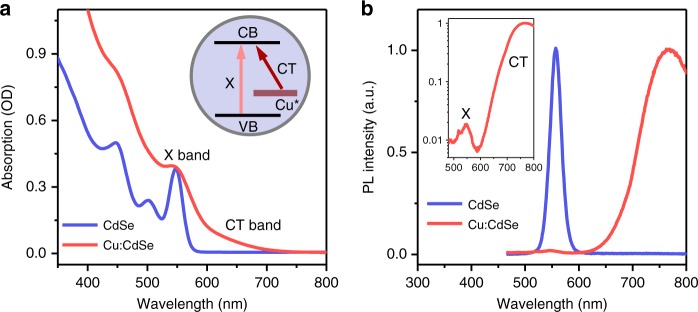
Optical properties of Cu-doped QDs. **a** Absorption spectra of CdSe (blue) and Cu:CdSe (red) QDs dispersed in heptane. Exciton (X) and charge-transfer (CT) bands are labeled, which are associated with transitions from the valence band (VB) edge and intragap copper state (Cu*), respectively, to the conduction band (CB) edge (inset). **b** Photoluminescence (PL) spectra of CdSe (blue) and Cu:CdSe (red) QDs excited at 400 nm. The latter is also plotted in logarithmic scale to show both X and CT band emissions for Cu:CdSe QDs (inset)

Absorption spectra of the doped and undoped QDs are displayed in Fig. 2a. The lowest energy excitonic transition bands (labeled as X band) of both samples are situated at ~550 nm. The X band is broadened in doped QDs as compared to undoped ones, the reason for which remains to be elucidated as the size distributions of the doped and undoped QDs are similar (Supplementary Fig. 1). A major difference between the two samples is the appearance of an absorption tail extending to ~700 nm for Cu:CdSe QDs. This feature cannot arise from scattering as the QDs were well-dispersed in heptane. A similar feature was extensively reported for Cu:QDs^27^, including ref. ^37^ from which we followed the synthetic procedures, and has been assigned to a charge-transfer type transition (CT band) from the intragap Cu* states to the conduction band (CB) edge of QDs (see Fig. 2a inset)^27,30^. Such an assignment is further confirmed by the appearance of the CT feature on emission and transient absorption spectra to be shown below.

Figure 2b shows the photoluminescence (PL) spectra of CdSe and Cu:CdSe QDs; the former exhibits an X band emission at ~560 nm, whereas the latter is dominated by a broad, red-shifted emission centered at ~780 nm which can be assigned to the CT band (i.e., recombination between an electron in the CB of QDs and a hole captured by the Cu* states). Nonetheless, a logarithmic plot of the PL spectrum of Cu:CdSe QDs (Fig. 2b inset) shows that it also has a weak X band emission. We note that the relative intensity of the X and CT band emissions can be controlled by the amounts of Cu incorporated into QDs, with higher Cu concentrations favoring relatively stronger CT band emissions^37^. This dual-emissive behavior has been assigned to either a competition between X and CT band emissions^29^ or a co-existence of doped and undoped QDs in the ensemble^27,37^. We show below using time-resolved PL and transient absorption (TA) spectroscopy that the latter explanation applies to our samples. Accordingly, here we adopted a relatively high Cu concentration in order to minimize the influence of undoped QDs on the examination of hot electron dynamics in Cu-doped samples.

### Femtosecond hole capturing in Cu-doped QDs

**Fig. 3:**
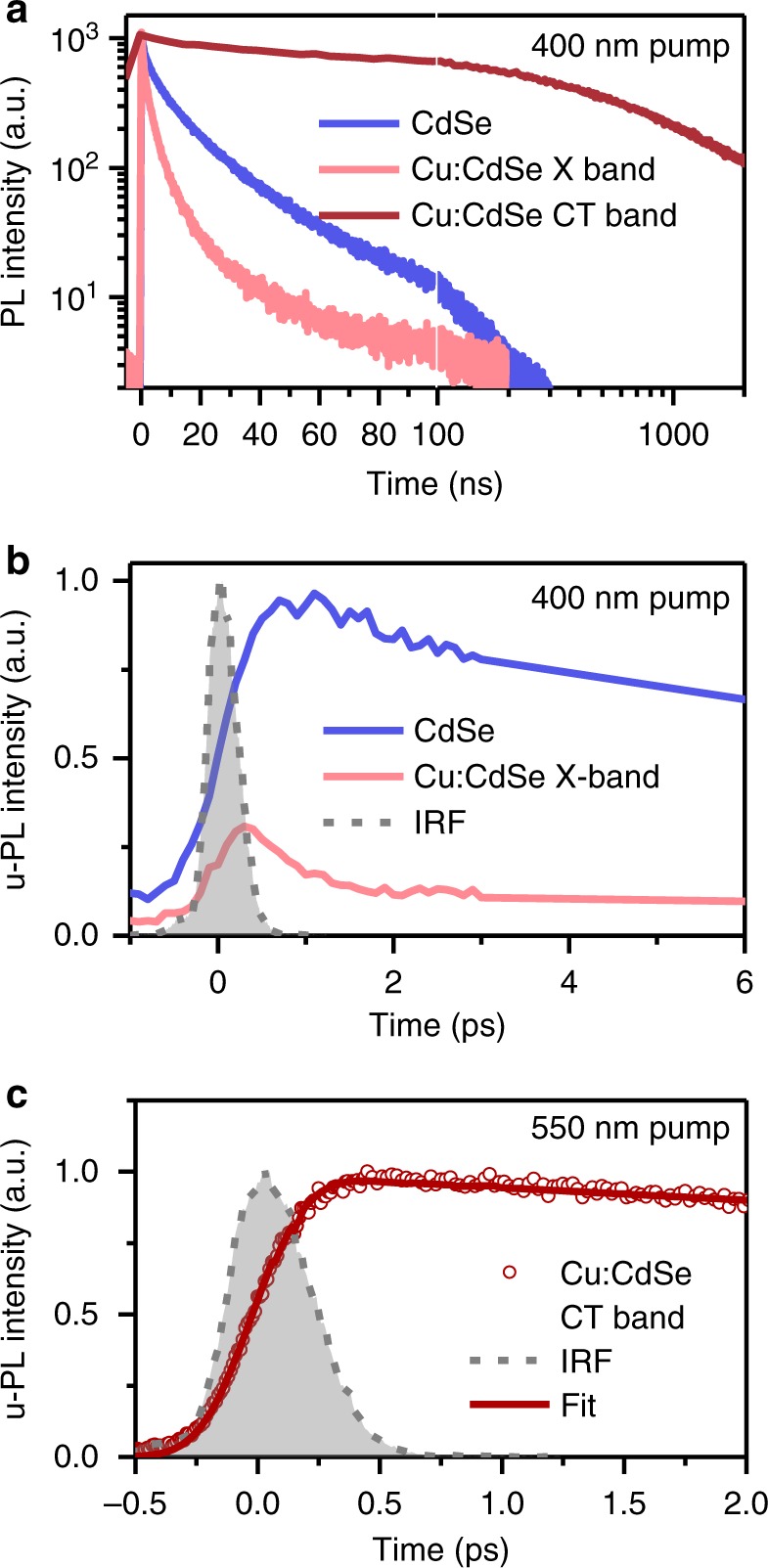
Femtosecond hole capturing in Cu-doped QDs. **a** Time-resolved PL kinetics for CdSe QDs (blue) and X (light red) and CT (dark red) bands in Cu:CdSe QDs. The first 100 ns is in linear scale while the rest is in logarithmic scale. **b** u-PL kinetics for CdSe QDs (blue) and X band in Cu:CdSe QDs (light red) measured by femtosecond upconversion excited at 400 nm. The gray dashed line (shaded) is the IRF of the system (390 fs). **c** u-PL kinetics of the CT band of Cu:CdSe QDs excited at 550 nm (dark red circles) and its fit (dark red solid line) by convoluting with the IRF

Figure 3a shows PL kinetics of QDs measured using time-correlated single-photon counting (TCSPC; see Methods for details). The CT band of Cu:CdSe QDs has a lifetime on the order of μs, consistent with the reduced wavefunction overlap between the delocalized electron and the Cu-localized hole. In contrast, the X band emission of Cu:CdSe QDs has a much shorter lifetime, with a very fast component within the instrument response function (IRF) of the set-up (~200 ps) and other slower components that are similar to those of undoped CdSe QDs (Fig. 3a). This similarity suggests the X band emission observed for Cu:CdSe QDs in Fig. 2b is from a subset of undoped dots in the ensemble.

In order to resolve the fast component for the X band emission, we performed femtosecond PL upconversion measurements (u-PL; see Methods for details). As shown in Fig. 3b, for CdSe and Cu:CdSe QDs with the same concentration and measured under the same conditions (excited with 400 nm pulses), the latter has a much lower initial signal amplitude than the former. This implies the majority of the X band emission of Cu:CdSe QDs either never forms or already decays within the IRF of the u-PL setup (~390 fs), suggesting that hole capturing time from the valance band (VB) to Cu* states is ≪390 fs.

We also measured the u-PL kinetics for the CT band of Cu:CdSe QDs excited with 550 nm pump pluses which resonantly created band edge QD excitons. In this case, the formation kinetics of the CT band emission should reflect the kinetics of hole transfer from QD band edges to Cu* states. As shown in Fig. 3c, the formation is essentially IRF-limited. A single exponential fit convoluting with the IRF reveals a formation time constant ≪390 fs. This femtosecond hole capturing process should outcompete the Auger-type electron-to-hole energy transfer occurring on a sub-ps timescale. Although not reported in refs. ^35,36^, we suspect the QDs used there might have a much slower hole capturing rate, thus allowing hot electron relaxation via energy transfer to the hole. This is likely due to the low Cu concentration in the QDs used there, because the hole transfer rate should roughly scale with the number of available Cu dopants inside the QD.

### Long-lived hot electrons in Cu-doped QDs

**Fig. 4:**
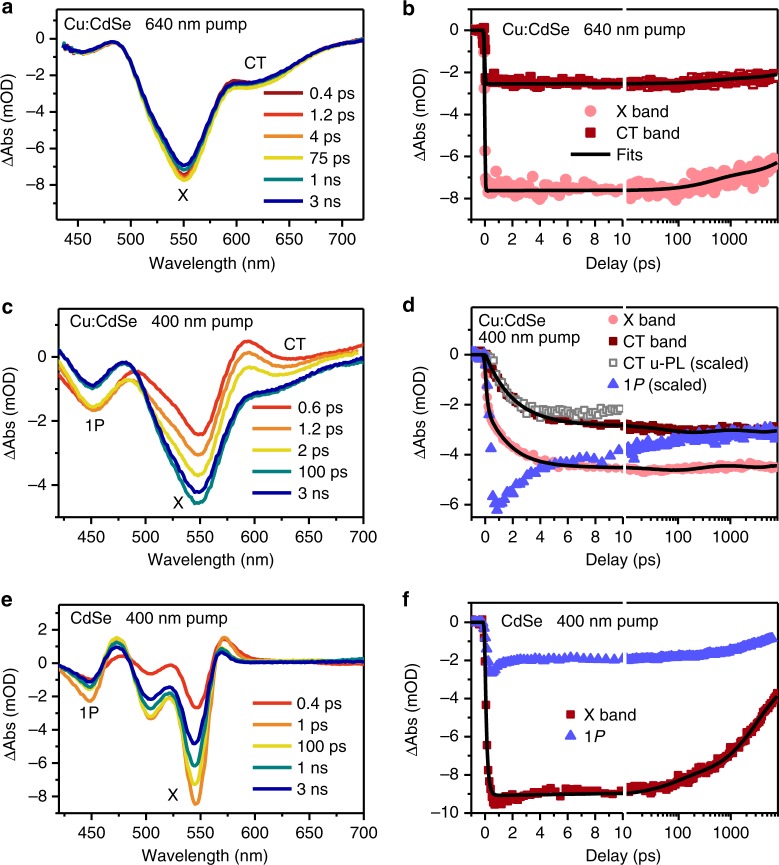
Hot electron relaxation in Cu-doped QDs. **a** TA spectra of Cu:CdSe QDs probed at indicated time delays following the excitation by a 640-nm pulse. **b** TA kinetics probed at the X (light red circles) and CT (dark red squares) band bleaches following the excitation by a 640-nm pulse. **c** TA spectra of Cu:CdSe QDs probed at indicated time delays following the excitation by a 400-nm pulse. **d** TA kinetics probed at the X (light red circles), CT (dark red squares), and 1*P* (blue triangles) band bleaches following the excitation by a 400-nm pulse. Note that the CT and 1*P* band signals have been amplified by factors of 3 and 4, respectively. Also shown is the u-PL kinetics of CT band emission (scaled and inverted; gray open squares) measured under 400 nm excitation with <*N*>~ 4. **e**, **f** Similar plots as (**c**, **d**) for undoped CdSe QDs

In order to directly observe the effect of ultrafast hole capturing on hot electron cooling, we performed femtosecond transient absorption (TA) measurements (See Methods for details). First, we excited Cu:CdSe QDs using a 640-nm pump pulse, which resonantly addressed the CT transition from Cu* states to 1*S*_e_ state in the CB (Fig. 4a). This excitation creates bleaches of not only CT band (centered at ~630 nm) but also the X band (at ~550 nm). As shown in Supplementary Fig. 2, both bleach features can be completely removed with the addition of molecular electron acceptors, indicating both are dominated by the state filling effects of electrons rather than holes and more specifically, 1*S*_*e*_ state electrons, which is consistent with previous assignments for undoped II–VI group QDs^38^. Note that additional TA features in the <480 nm range can be assigned to Stark-effect-type signals of higher-lying excitonic bands arising from exciton–exciton Coulomb interactions^38,39^. Because of the resonant excitation, both X and CT band bleaches formed within the IRF (~90 fs) of our TA setup (Fig. 4b). Both features are long-lived, showing <15% decay within 8 ns (Fig. 4b), consistent with the μs-scale CT band emission observed in Fig. 3a. This also suggests our experiments are performed under single-exciton-dominated conditions (average exciton number per QD, < *N* > , < 0.1; see Supplementary Fig. 3 and Supplementary Note 1 for details) which exclude the complication from multi-exciton effects. Note that multi-exciton effects can be observed at very low exciton occupancies (<*N*>~0.05) in single dot PL lifetime measurements^40^. However, at the ensemble level the possibility of finding QDs excited with multi-excitons (*N* ≥ 2) is ~0.12% with <*N*>~0.05, estimated using a Poisson statistics^39,41,42^, which should be negligible for the ensemble kinetic traces.

Next, Cu:CdSe QDs were excited with a 400-nm pump pulse which is slightly above the energy of the 1 *P* exciton transition according to absorption spectra in Fig. 2a. The TA spectra also show bleaches of both X and CT bands (Fig. 4c) and some derivative-like features in the <480-nm range. Interestingly, however, the formation rates for X and CT bands are very slow. As shown in Fig. 4d, the signal amplitudes of X and CT bands continue growing until 100 s of ps. As the formation kinetics of X and CT bands is a measure of the electron cooling kinetics from 1*P*_*e*_ to 1*S*_*e*_ level, this result implies long-lived 1*P*_*e*_ electrons in Cu:CdSe QDs. This is in stark contrast to the X band kinetics in undoped CdSe QDs (Fig. 4e, f), which reaches its maximum at ~0.5 ps and starts to decay (Fig. 4f); it then shows ~50% decay within 8 ns due to radiative and/or nonradiative recombination of band edge 1*S*_*e*_ electrons. This comparison indicates the presence of a phonon bottleneck in Cu:CdSe QDs.

Long-lived 1*P*_*e*_ hot electrons in Cu:CdSe can also be directly observed on the 1*P* exciton bleach feature on TA spectra. As shown in Fig. 4d, TA kinetics probed near 450 nm, which contains some contributions from 1*P*_*e*_ electrons, exhibits slow decay components extending beyond 100 s of ps that are complementary to the slow formation components of the X and CT band kinetics (Fig. 4d). Such slow decay components are absent in undoped QDs (Fig. 4f), further confirming long-lived 1*P*_*e*_ hot electrons in Cu:CdSe QDs. The long-lived components beyond ns timescale probed at 450 nm result from the contributions of derivative-like features arising from Coulombic interactions mentioned above.

The slow hot electron cooling should also be manifested on the growth kinetics of the CT band emission in Cu:CdSe QDs. Figure 4d shows the u-PL kinetics (inverted and scaled) measured for the CT band with <*N*> ~4 (excited at 400 nm); although it shows some decay at >5 ps due to multi-exciton Auger recombination, it agrees well with the formation kinetics of the X band TA signal within 5 ps. Such an agreement further confirms slow hot electron relaxation in our Cu:CdSe QDs. Note that high excitation densities were used for u-PL measurements of the CT band emission due to its long lifetime and hence very low u-PL photon counts.

We performed quantitative analysis for hot electron relaxation using the X and CT band TA signals. Scrutiny of the X and CT band kinetics of Cu:CdSe QDs in Fig. 4d reveals differences between them; the former has a portion of signal amplitude forming on sub-ps scale, which is indicative of a subset of undoped QDs in the Cu:CdSe sample. To verify it, we compare the TA spectra of Cu:CdSe QDs created by 400 nm and 640 nm excitations in Supplementary Fig. 4. When normalized at the CT band signal, the 400-nm excited spectrum has a higher X band signal than the 640-nm excited one. As the latter only probes doped QDs, this difference proves the existence of undoped QDs in the ensemble that are also excited by the 400-nm pulse. We use the ratio between X band bleach amplitudes under 640 nm and 400 nm excitations as a measure of the percentage of doped QDs in the Cu:CdSe QDs, which is 67 ± 3% according to Supplementary Fig. 4. The rest 33 ± 3% is contributed by undoped QDs in the ensemble. This population ratio can indeed successfully account for the difference between X and CT band kinetics. As shown in Supplementary Fig. 4, when the CT band kinetics is down-shifted by an amplitude corresponding to the undoped population (33%), it matches the X band kinetics well.

With the doped and undoped populations in the Cu:CdSe sample resolved, we fit the kinetics in Fig. 4d using multi-exponential functions; see Supplementary Note 2 for details. The kinetics of the X band of CdSe QDs can be fitted with a rising time of 0.25 ± 0.05 ps, consistent with the sub-ps hot electron cooling time in typical QDs, and some decay components spanning ps to ns timescale (Supplementary Table 1) accounting for radiative and/or nonradiative recombination of band edge 1*S*_*e*_ electrons. The kinetics of the CT band of Cu:CdSe QDs is fitted to a two-exponential formation followed by a two-exponential decay. The fitted formation time constants (and corresponding relative amplitudes) are 2.10 ± 0.10 ps (90%) and 67.4 ± 3.6 ps (10%); See Supplementary Table 2. The kinetics of the X band of Cu:CdSe QDs can be fitted as a superposition of their CT band kinetics and the X band kinetics of undoped QDs scaled by appropriate factors. The amplitude-weighted average time constant for hot electron relaxation in Cu:CdSe QDs is 8.6 ± 0.4 ps, which is ~34-fold longer than the time constant in undoped QDs (0.25 ps).

In order to confirm that the long-lived hot electron is a general phenomenon rather than a coincidental observation for a specific sample at a specific excitation wavelength, we have performed TA experiments using different excitation wavelengths and on different samples. As shown in Supplementary Fig. 5, 340 nm excitation of the sample used here also shows long-lived hot electrons. In addition, both 340 nm and 400 nm excitations of a different-size Cu:CdSe QD sample (with the X band at 570 nm) leads to similar observations (Supplementary Figs. 6–8).

The heterogeneity in the hot electron lifetime of Cu:CdSe QDs likely arises from a distribution of coupling strength between CB electrons and Cu*-localized holes which can ultimately be traced to spatial distribution of Cu-dopants inside the QD. Dopants located far away from the QD center presumably couple more weakly to the delocalized CB electrons, leading to longer-lived hot electrons. Other effects such as energy transfer to surface-bound ligands might also be partially responsible for the heterogeneity of the hot electron lifetime.

Although we have attributed the long-lived hot electrons in Cu:CdSe QDs mainly to the suppression of Auger-type, electron-to-hole energy transfer, hole capturing by Cu-dopants likely also suppresses another hot electron relaxation mechanism, that is, nonadiabatic interaction with surface ligands^20,23,43^. Specifically, the carrier wavefunctions of strong-confined QDs spread onto QD surfaces and interact with the nuclei coordinates of surface ligands, which can induce nonadiabatic transitions between quantum-confined states. Thus, even if the electron-to-hole energy transfer pathway is shut down, hot electrons in colloidal QDs can still rapidly relax via this nonadiabatic mechanism. In our Cu:CdSe QDs, this mechanism could be suppressed because the Coulombic attraction between the Cu-localized hole and the conduction band electron should result in shrinkage of the electron wavefunction, reducing the wavefunction fraction on QD surfaces and hence suppressing its nonadiabatic interaction with surface ligands. Suppression of both electron-to-hole energy transfer and nonadiabatic interactions enables the observation of a phonon bottleneck in Cu:CdSe QDs.

### Comparison to lead halide perovskites

As we mentioned in the introduction, both colloidal QDs and lead halide perovskites have been extensively studied under the context of hot carrier relaxation. Recently, lead halide perovskites attract more attention than QDs for at least two reasons: (i) Many years of research into QDs has only led to limited success in terms of prolonging their hot carrier lifetime^21^; (ii) Some recent studies reported spectroscopic signature for long-lived hot carriers (100 s of ps) in lead halide perovskites^4–6^. Now that we have prolonged the hot electron lifetime in colloidal CdSe QDs by orders of magnitudes, it is interesting to compare the hot electron lifetime in Cu:CdSe QDs with those reported for lead halide perovskite nanocrystals (NCs) and bulk materials^4,6–12^.

Here we focus on hot carrier cooling dynamics under low excitation densities. Effects such as Auger heating^44^ and hot phonon bottleneck^12^ that can further slow down hot carrier cooling at higher excitation densities are beyond the scope of this work. Note the difference between the concepts of hot phonon bottleneck and phonon bottleneck. The former is a result of efficient electron–phonon scattering leading to a fast build-up of a large population of, for example, hot LO phonons; if these hot LO phonons lose their energy to acoustic phonons with a relatively slower rate, they in turn heat up the electrons and thus slow down hot electron relaxation. In contrast, phonon bottleneck refers to very weak electron–phonon scattering which can slow down hot electron cooling even at vanishing excitation densities. Arguably, phonon bottleneck is more relevant to real-life solar radiation conditions, whereas hot phonon bottleneck would typically require concentrated sunlight.

**Fig. 5:**
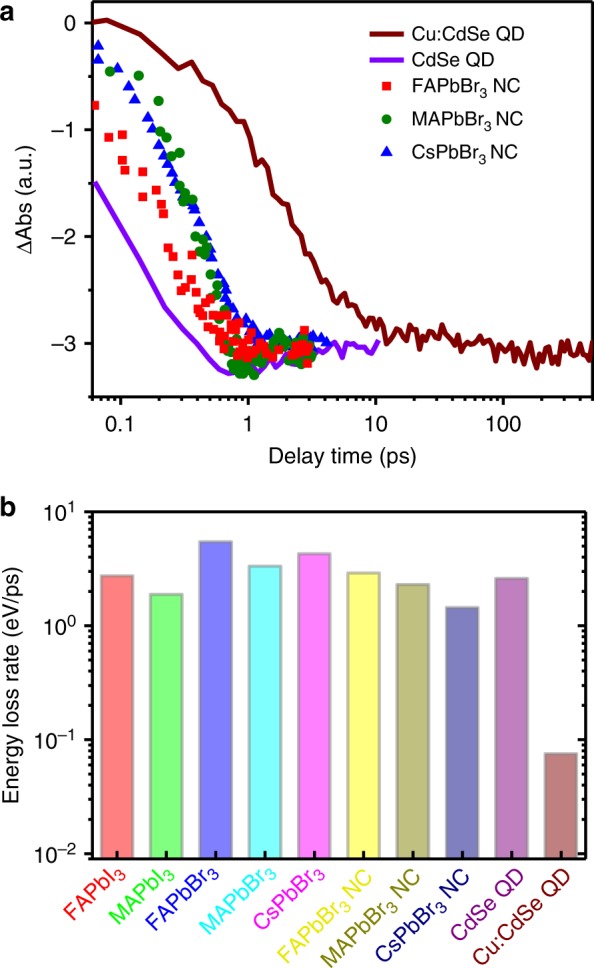
Comparison to lead halide perovskites. **a** Exciton bleach formation kinetics of CdSe (purple line) and Cu:CdSe QDs (wine line) in this study (400 nm pump) and FAPbBr_3_ (red squares), MAPbBr_3_ (green circles), and CsPbBr_3_ (blue triangles) NCs adapted from ref. ^8^ (350 nm pump). All the kinetics were measured under sub-single-exciton conditions. **b** Hot carrier energy loss rates calculated for FAPbI_3_, MAPbI_3_, FAPbBr_3_, MAPbBr_3_, and CsPbBr_3_ bulk films, FAPbBr_3_, MAPbBr_3_, and CsPbBr_3_ NCs, and CdSe (purple) and Cu:CdSe QDs (wine). The data for perovskite bulk films and NCs are adapted from refs. ^7,8^ respectively

Figure 5a shows the comparison of exciton bleach formation kinetics in CdSe and Cu:CdSe QDs in this study (400 nm pump) and FAPbBr_3_, MAPbBr_3_, and CsPbBr_3_ NCs measured under sub-single-exciton conditions (350 nm pump) adapted from ref. ^8^ While lead bromide perovskite NCs with various cation types show slower hot carrier cooling than undoped CdSe QDs, they display much (at least one order of magnitude) faster cooling than our Cu:CdSe QDs.

In order to quantitatively compare hot carrier cooling in various systems, it is necessary to account for the excessive energies of the hot carriers above their band edges (Δ*E*). Δ*E* can be estimated from the pump photon energies and the band structures of the investigated materials; see Supplementary Note 3 and Supplementary Table 3 for details. We can then calculate the hot carrier energy loss rate (d*E/*d*t*) using: d*E/*d*t* = Δ*E/τ*, where *τ* is the hot carrier relaxation time constant under very weak excitation densities. We made calculations for not only lead bromide perovskite NCs reported in ref. ^8^ but also various lead halide perovskite bulk films reported in ref. ^7^ The results are tabulated in Supplementary Table 3 and plotted in Fig. 5b. Clearly, d*E/*d*t* of our Cu:CdSe QDs is much slower than that of all perovskite materials being compared. Specifically, the slowest hot carrier energy loss rate among perovskites is 1.44 eV/ps measured for CsPbBr_3_ NCs, whereas the hot electron energy loss rate of Cu:CdSe QDs is almost 20-fold slower than this value (0.075 eV/ps) because of a phonon bottleneck.

### Implication for devices

Although we have observed a hot electron lifetime of ~8.6 ps for Cu:CdSe QDs in solution, for potential applications such as solar cells QDs are often in solid state. As such, it is important to examine whether incorporation into solid state films could introduce additional relaxation pathways affecting the hot electron lifetime. To this end, we also made measurements for QDs in densely packed films and QDs adsorbed onto mesoporous metal oxide films; see Supplementary Figs. 9 and 10, respectively, for the data and Methods for the fabrication of these samples. In both cases, if no additional charge-transfer pathways are introduced, the hot electron relaxation kinetics remains largely unaffected in the solid state, suggesting that the long-lived electrons should still be available when Cu:CdSe QDs are incorporated into devices.

Densely packed films correspond to the situation of heterojunction solar cells using QD solids. In this case, the hot electrons need to travel through the film to reach the heterojunction interface for hot electron extraction, which is very challenging considering the relatively low carrier mobility in QD solids as compared to bulk semiconductors^45^. In this sense, lead halide perovskites should perform better, despite that our comparison above indicate they have faster hot carrier energy loss rates than our Cu:CdSe QDs, as Huang et al. has reported a quasi-ballistic transport distance of up to 230 nm for hot carriers in MAPbI_3_ films^6^.

On the other hand, QDs adsorbed onto mesoporous metal oxide films are well suited for sensitized solar cells, where they are in direct contact with metal oxides and no carrier transport is required. Kamat et al. reported an electron transfer (ET) time constant of ~3.6 ps from CdSe QDs (diameter ~2.8 nm) to mesoporous SnO_2_ films^46^, which should be fast enough to harvest hot electrons from Cu:CdSe QDs. If the surface of QDs were treated with short chain ligands to enable a strong electronic coupling between QDs and metal oxides, an even faster ET time and hence higher hot ET yield could be expected, as demonstrated by Zhu et al. for the case of PbSe QDs on TiO_2_ single crystals^47^. Our preliminary measurements on Cu:CdSe QDs on mesoporous TiO_2_ films indeed show some evidence for hot electron extraction (Supplementary Fig. 10 and Supplementary Note 4). However, because of the lack of a direct observation of electrons injected into TiO_2_ conduction band, we do not make any conclusive argument here and future efforts are needed for convincing demonstration of hot electron extraction.

## Discussion

In conclusion, we have studied hot electron relaxation in Cu-doped CdSe QDs in comparison with undoped ones. We found that the simple Cu-doping procedure could prolong the lifetime of 1*P*_*e*_ hot electrons from ~0.25 ps in undoped QDs to ~8.6 ps in doped QDs. Ultrafast spectroscopic analysis indicated that this slow hot electron cooling behavior was likely enabled by the ultrafast capturing (≪ 390 fs) of photogenerated holes by Cu-related intragap states. This hole capturing effectively decouples electrons from holes and thus inhibits hot electron relaxation via Auger-type electron-to-hole energy transfer in CdSe QDs. It also likely leads to electron wavefunction shrinkage, suppressing hot electron relaxation via nonadiabatic transitions induced by interacting with nuclei coordinates of surface ligands. These two effects induced by hole capturing enforce a phonon bottleneck for hot electrons. As a result, the hot electron lifetime in Cu-doped CdSe QDs not only is much longer than undoped QDs but also compares favorably with lead halide perovskite materials extensively studied of late for hot carrier applications. Quantitatively, the hot electron energy loss rate in Cu-doped CdSe QDs is 0.075 eV/ps, which is more than 20-fold slower than all perovskite nanocrystals and films measured under low excitation power densities. This comparison suggests a novel opportunity for hot carrier related solar energy applications using Cu-doped colloidal QDs.

## Methods

### Synthesis of Cu-doped and undoped QDs

The Undoped CdSe QDs were synthesized using a previously reported “heat-up” method^48^. In a typical synthesis, a mixture of 0.135 g of cadmium oleate, 0.022 g of selenium dioxide, and 8.8 g of ODE were loaded into a three-neck flask and was degassed under vacuum at 55 °C for 45 min. The mixture was then heated at 235 °C under N_2_ atmosphere and aliquots were taken from the mixture to monitor the growth of QDs. When the lowest energy peak of QDs reached 550 nm, the reaction was stopped and QDs were precipitated from the mixture by adding ethanol. The precipitants were re-dispersed in toluene and precipitated by ethanol. This was repeated for three times and the final products were dispersed in heptane for optical measurements.

Cu:CdSe QDs were synthesized by adding a copper precursor solution into a solution of preformed CdSe QDs (without washing)^37^. In brief, the copper precursor solution was prepared by mixing 34.6 mg of copper iodide (CuI) with 2.4 g of octadecene (ODE) and was purged with nitrogen before adding 0.1 mL of trioctylphosphine (TOP). The mixture was sonicated for 45 min to produce a colorless solution. This precursor solution was injected into a CdSe QD solution maintained at 235 °C, leading to an immediate color change of the QD solution. The reaction was continued for 20 min before being quenched by removing the heating mantle and adding ethanol to precipitate out the doped QDs. The doped QDs were washed by three times and the final products were dispersed in heptane for optical measurements.

Densely-packed QD films were prepared by drop a highly concentrated QD hexane solution onto a glass substrate followed by spin-coating at 2000 rpm. The films were then transferred to a nitrogen-filled glove box and sealed before being transferred out of the box for optical measurements. QD-sensitized mesoporous metal oxide films were prepared by immersing semitransparent TiO_2_ or ZrO_2_ films into a concentrated QD hexane solution in the glove box for 24 h. The films were rinsed by hexane to remove unattached QDs and were sealed in airtight cuvettes before being transferred out of the box for optical measurements.

### Time-resolved PL

The femtosecond PL upconversion (u-PL) experiments were performed on a Chimera spectrometer (Light conversion) using a Pharos laser (1030 nm,100 kHz, 230 fs pulse-duration; Light conversion) as the excitation and gate sources. One part of the Pharos output was used to pump an optical parametric amplifier (OPA; TOPAS) to generate the wavelength-tunable excitation pulses, while the other was used as the gate pulse. The light emitted by the sample was collected by lens and focused into a BBO crystal together with the gate pulse to generate the up-converted signal via sum frequency generation (SFG). The signal photons were focused onto the entrance slit of a monochromator and detected by the spectrometer. The time-resolved decay curve was obtained by delaying the gate pulse with respect to the pump pulse using a mechanical delay stage. Time-correlated single-photon counting (TCSPC) measurements use the same excitation source and detector as the u-PL set-up. The samples were placed in 1 mm quartz cuvettes and were vigorously stirred in all the measurements in order to minimize photocharging or damaging of the samples.

### Transient absorption

Femtosecond pump-probe TA measurements were performed using a regenerative amplified Ti:sapphire laser system (Coherent; 800 nm, 70 fs, 6 mJ/pulse, and 1 kHz repetition rate) as the laser source and a femto-TA100 spectrometer (Time-Tech Spectra). Briefly, the 800 nm output pulse from the regenerative amplifier was split in two parts with a 50% beam splitter. The transmitted part was used to pump an OPA which generated a wavelength-tunable laser pulse from 250 nm to 2.5 μm as pump beam. The reflected 800 nm beam was split again into two parts. One part was attenuated with a neutral-density filter and focused into a 2-mm-thick sapphire or CaF_2_ window to generate a white light continuum (WLC) used for probe beam. In general, CaF_2_ window gives higher light quality in the <450-nm range, which is more suited for the study of higher-energy features (such as 1*P*) of QDs. The probe beam was focused with an Al parabolic reflector onto the sample. After the sample, the probe beam was collimated and then focused into a fiber-coupled spectrometer with CMOS sensors and detected at a frequency of 1 kHz. The intensity of the pump pulse used in the experiment was controlled by a variable neutral-density filter wheel. The delay between the pump and probe pulses was controlled by a motorized delay stage. The pump pulses were chopped by a synchronized chopper at 500 Hz and the absorbance change was calculated with two adjacent probe pulses (pump-blocked and pump-unblocked). The samples were placed in 1 mm quartz cuvettes and were vigorously stirred in all the measurements.

## Supplementary information

Supplementary Information

Peer Review File

## Data Availability

The experiment data that support the findings of this study are available from the corresponding author upon reasonable request.
