# Supplementary material for: Observation of a phonon bottleneck in copper-doped colloidal quantum dots
Source: Nat Commun. 2019 Oct 4;10:4532. doi: 10.1038/s41467-019-12558-y (PMC6778069; doi:10.1038/s41467-019-12558-y)
Supplement: Supplementary file 1 — Supplementary Information [file 41467_2019_12558_MOESM1_ESM.pdf]

Reviewers' comments:

Reviewer #1 (Remarks to the Author):

In the manuscript presented here, Wang et al. investigate the effect of Cu-doping on the hot carrier cooling dynamics in CdSe QDs. Longer hot electron lifetimes are reported in the case of Cu-doping, which is attributed to suppressed Auger-type recombination due to hole capturing by the dopant atoms.
The manuscript is overall sound and the conclusions can be supported by the data, and the methods are detailed well, allowing other researchers to reproduce this data.

However, I present a few suggestions to improve the manuscript and its readability/flow.

1) On page 8, the authors claim that the low number of excitons <N> < 0.1 is sufficient to exclude effects caused by multi-excitons. I would strongly disagree with this statement as correlation studies on multi-excitons are generally performed well below this threshold. E.g. work by Bawendi shows up to triexciton emission at 0.05 excitations/pulse for CdSe nanoparticles. (Shulenberger et al. Nano Lett.2018,18,5153-5158). Appropriate literature on higher-order excitons should be cited and included in the introduction and discussion and the authors should ensure that they have no effects by higher order excitons.
2) Accordingly, Figure 3a must be changed to show a proper view of the X decay, a double-log plot or an inset would suffice.
3) Finally, I find the comparison to lead halide perovskites unnecessary and confusing. It does not appear to add to the manuscript and the abrupt topic change is hard to follow. As such, I would suggest to either remove this paragraph, as well as Figure 5 entirely from the manuscript or write a proper lead into this topic.

Reviewer #2 (Remarks to the Author):

Wang et al, reported an interesting phonon bottleneck in copper-doped colloidal quantum dots using transient absorption spectroscopic technique. It is much beneficial to harvest hot electrons for improving the efficiency of photovoltaic devices, thus is a hot research topic in the energy-related field. To see the phonon bottleneck in colloidal quantum dots, one needs to suppress all other competing relaxation processes in hot electron cooling, for example, electron-phonon scattering and electron-hole Auger relaxation. In this manuscript, the excited holes in Cu-doped CdSe QDs can be efficiently captured by Cu-introduced intragap states, which can facilitate to slow down the cooling processes of hot electrons. They observed a lifetime of ~8.6 ps for 1P hot electrons in Cu-doped QDs, which is much longer than that in pure CdSe QDs. Their reports are interesting and might be useful for potential applications in solar cells, however, some issues still need to be addressed.
Comment 1.
The lifetime of 1P hot electron in Cu-doped CdSe QDs derived from the experiments is ~8.6 ps, which is still efficient as compared with the charge transfer dynamics between QDs and metal oxides for extraction of hot electrons. Do the authors have any comments on how to extract such hot electrons for potential applications in solar cells.
Comment 2.
This phonon bottleneck behavior was observed for the samples well dispersed in solution. However, for PV applications, those QDs need to be transformed to solid state. In the solid state, extra efficient energy relaxation pathways can be also introduced. The authors should also investigate the ultrafast electron relaxation dynamics in Cu-doped QDs or Cu-doped QDs integrated to charge-transfer layers in the solid state.
Comment 3.
I suggest the authors can compare the hot electron relaxation dynamics and related lifetimes in the different materials systems and give some comments about hot-electron extraction for potential applications in solar cells.

Reviewer #3 (Remarks to the Author):

In this manuscript, Wu et al. report the hot electron relaxation dynamics in Cu doped CdSe quantum dots. Using the combination of ultrafast absorption and emission spectroscopy, the authors demonstrated that the presence of Cu in CdSe can significantly elongate hot electron cooling time, which can be attributed to the effective hole capturing by Cu, resulting weaker electron-hole coupling and enforcing photon bottleneck. In my opinion, this work is interesting and significant for two reasons: 1) the fundamental understanding of photon bottleneck is critical to resolve the Shockley-Queisser limit, which is anticipated to be the key limiting factor to further improve solar energy conversion efficiencies; 2) the authors have carried out a systematic studies combining careful experiment and thorough data analysis. I recommend publication in this journal if the authors can address the minor concern listed below.
1) The elongated hot electron lifetime has been reported before in Cu doped CdSe quantum dots as author stated in the manuscript (see line 71-73). Why much slower relaxation time was observed in this work than previous Cu doped CdSe systems? What are the key factors that lead to the difference of hole relaxation dynamics between Cu doped CdSe systems reported by different groups?

Reviewers’ comments in black; our responses in red and revisions in blue.

Reviewers' comments:

Reviewer #1 (Remarks to the Author):

In the manuscript presented here, Wang et al. investigate the effect of Cu-doping on the hot carrier cooling dynamics in CdSe QDs. Longer hot electron lifetimes are reported in the case of Cu-doping, which is attributed to suppressed Auger-type recombination due to hole capturing by the dopant atoms.
The manuscript is overall sound and the conclusions can be supported by the data, and the methods are detailed well, allowing other researchers to reproduce this data.
Response: We thank the reviewer very much for his/her kind comments. Below we provide our point-to-point responses and revisions to address his/her remaining concerns.

However, I present a few suggestions to improve the manuscript and its readability/flow.

1) On page 8, the authors claim that the low number of excitons <N> < 0.1 is sufficient to exclude effects caused by multi-excitons. I would strongly disagree with this statement as correlation studies on multi-excitons are generally performed well below this threshold. E.g. work by Bawendi shows up to triexciton emission at 0.05 excitations/pulse for CdSe nanoparticles. (Shulenberger et al. Nano Lett.2018,18,5153-5158). Appropriate literature on higher-order excitons should be cited and included in the introduction and discussion and the authors should ensure that they have no effects by higher order excitons.
Response: We thank the reviewer for reminding us the relevant papers on the exciton statistics in QDs. Indeed, in this Nano Lett. paper, the photon number resolved single dot studies are very sensitive to the number of excitations per QDs, and hence extremely low excitation densities were used.

However, our ensemble TA and PL kinetics measurements were not as sensitive to the number of excitations per QDs (<*N*>). The <*N*> value used in our experiment was ~0.05, as specified in Supplementary Fig. 3. According to the well-established Poisson statistics for photon absorption in QDs (when the excitation pulse, 400 nm, is far above the band edge), the possibility of finding QDs excited with more than 2 photons (*N* ⩾ 2) is: 1 - exp(-<*N*>) - <*N*>*exp(-<*N*>) = 0.12%. In our ensemble measurements, this percentage barely affects our kinetic traces and we can conclude the complication from multi-exciton effects is negligible. In fact, when we re-plot the TA kinetics under different pump fluences by appropriately scaling their early-time signals (Fig. R1), we find that the hot electron relaxation kinetics remains similar for fluences below 28 μJ/cm^2 (<*N*> = 0.2).

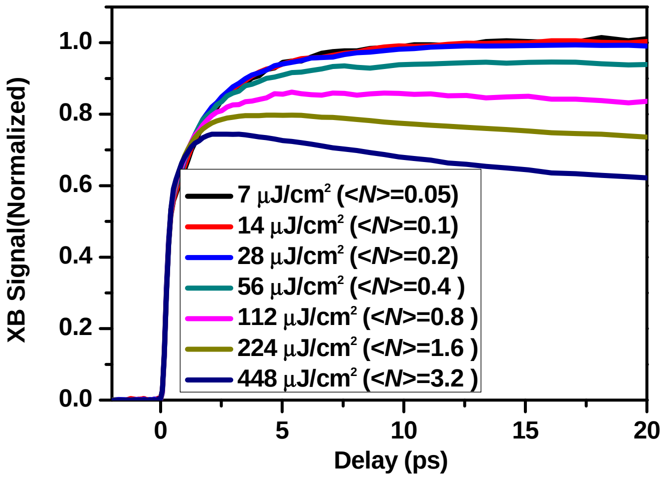

Fig. R1. TA kinetic traces under pump fluences from 7 μJ/cm^2 to 448 μJ/cm^2 (<*N*> from 0.05 to 3.2), appropriately rescaled to shown the effects of multi-exciton excitation on hot electron relaxation kinetics in the first 20 ps.

Revision: In the paragraph the reviewer referred to, we add the following contents to discuss the possibility of multi-excitons effects by citing relevant literature papers including the one mentioned by the reviewer:

"Note that multi-exciton effects can be observed at very low exciton occupancies (<*N*> ~ 0.05) in single dot PL lifetime measurements.[40] However, at the ensemble level the possibility of finding QDs excited with multi-excitons (*N* ⩾ 2) is ~0.12% with <*N*> ~ 0.05, estimated using a Poisson statistics,[39,41,42] which should be negligible for the ensemble kinetic traces."

In Supplementary Fig. 3, we add the figure shown above as the Supplementary Fig. 3c to ensure that the hot electron relaxation kinetics we measured is not affected by multi-exciton effects.

2) Accordingly, Figure 3a must be changed to show a proper view of the X decay, a double-log plot or an inset would suffice.

Response: We thank the reviewer for this useful suggestion.

Revision: We have now revised the figure (Fig. R2) to use a linear plot in the first 100 ns (to better show the X band PL decay) and a log plot until 2000 ns (to better show the CT band PL decay).

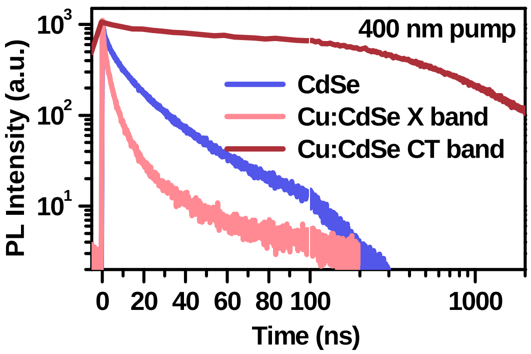

Fig. R2. Time-resolved PL kinetics for CdSe QDs (blue) and X (light red) and CT (dark red) bands in Cu:CdSe QDs. The first 100 ns is in linear scale while the rest is in logarithmic scale.

3) Finally, I find the comparison to lead halide perovskites unnecessary and confusing. It does not appear to add to the manuscript and the abrupt topic change is hard to follow. As such, I would suggest to either remove this paragraph, as well as Figure 5 entirely from the manuscript or write a proper lead into this topic.

Response: We understand the reviewer's concern that the perovskite part is not tightly related to the Cu-doped QDs studied in this paper. The reason why we want to make this discussion is that both class of materials, lead halide perovskites and colloidal QDs, have been extensively studied under the context of hot carrier relaxation. It is thus informative to make a comparison between them to give the readers a general overview of the current status on this topic.

Revision: We add the following contents to the beginning of the section of comparison to lead halide perovskites in order to improve the flow of the manuscript:
"As we mentioned in the introduction, both colloidal QDs and lead halide perovskites have been extensively studied under the context of hot carrier relaxation. Recently, lead halide perovskites attract more attention than QDs for at least two reasons: i) Many years of research into QDs has only led to limited success in terms of prolonging their hot carrier lifetime;[21] ii) Some recent studies reported spectroscopic signature for long-lived hot carriers (100s of ps) in lead halide perovskites.[4-6] Now that we have prolonged the hot electron lifetime in colloidal CdSe QDs by orders of magnitudes, it is interesting to compare the hot electron lifetime in Cu:CdSe QDs with those reported for lead halide perovskite nanocrystals (NCs) and bulk materials.[4,6-12]"

Reviewer #2 (Remarks to the Author):

Wang et al, reported an interesting phonon bottleneck in copper-doped colloidal quantum dots using transient absorption spectroscopic technique. It is much beneficial to harvest hot electrons for improving the efficiency of photovoltaic devices, thus is a hot research topic in the energy-related field. To see the phonon bottleneck in

colloidal quantum dots, one needs to suppress all other competing relaxation processes in hot electron cooling, for example, electron-phonon scattering and electron-hole Auger relaxation. In this manuscript, the excited holes in Cu-doped CdSe QDs can be efficiently captured by Cu-introduced intragap states, which can facilitate to slow down the cooling processes of hot electrons. They observed a lifetime of ~8.6 ps for 1P hot electrons in Cu-doped QDs, which is much longer than that in pure CdSe QDs. Their reports are interesting and might be useful for potential applications in solar cells, however, some issues still need to be addressed.

Response: We thank the reviewer very much for his/her kind comments. And we hope that our responses and revisions shown below can address the issues raised by the reviewer.

Comment 1.

The lifetime of 1P hot electron in Cu-doped CdSe QDs derived from the experiments is ~8.6 ps, which is still efficient as compared with the charge transfer dynamics between QDs and metal oxides for extraction of hot electrons. Do the authors have any comments on how to extract such hot electrons for potential applications in solar cells.

Response: We agree with the reviewer that the hot electron lifetime might still be shorter than the time constants of electron transfer (ET) from QDs to electron acceptors for real applications in solar cells. But prolonging the hot electron lifetime from a few 100s fs to 8.6 ps should enable a much better opportunity for hot electron extraction. For example, Kamat and coworkers reported an ET time constant of ~3.6 ps from CdSe QDs (diameter ~2.8 nm) to mesoporous SnO_2 films (*Proc. Natl. Acad. Sci.* **2011**, *108*, 29-34); Zhu and coworkers reported an hot ET time constant of <50 fs between strongly-coupled PbSe QDs and single-crystalline TiO_2 (*Science* **2010**, *328*, 1543-1547). Thus, in solar cells based on QD-sensitized metal oxide electrodes, we could expect that hot electron extraction might indeed be feasible. For more details, please see our response to your Comment 2 below.

Comment 2.

This phonon bottleneck behavior was observed for the samples well dispersed in solution. However, for PV applications, those QDs need to be transformed to solid state. In the solid state, extra efficient energy relaxation pathways can be also introduced. The authors should also investigate the ultrafast electron relaxation dynamics in Cu-doped QDs or Cu-doped QDs integrated to charge-transfer layers in the solid state.

Response: We thank the reviewer for the suggestion of measuring hot electron dynamics in the solid state. Previously, we didn't make the measurements because the paper was intended for a proof-of-concept demonstration of a system with long-lived hot electrons. We agree with the reviewer that eventually these QDs need to be incorporated into solid state for real device applications. As such, we have now made measurements for QDs in densely-packed solid state films and QDs absorbed onto mesoporous metal oxide films. The former case corresponds to the situation of

heterojunction solar cells using QD solids, whereas the latter is directly related to QD-sensitized solar cells.

In the case of densely-packed QD solid films prepared by spin-coating, we find that the hot electron relaxation kinetics (probed at the CT band) is almost the same as that of solution samples (Fig. R3), suggesting that the extra energy relaxation pathways introduced in the solid state (such as surface trap states) does not notably affect hot electron relaxation. However, in QD solid solar cells the hot electrons need to travel through the film to reach the heterojunction interface for hot electron extraction, which is very challenging considering the relatively low carrier mobility in QD solids as compared to bulk semiconductors. As such, a more realistic scheme would be to implement QDs into sensitized solar cells where all the QDs are in direct contact with metal oxides and only interfacial ET matters.

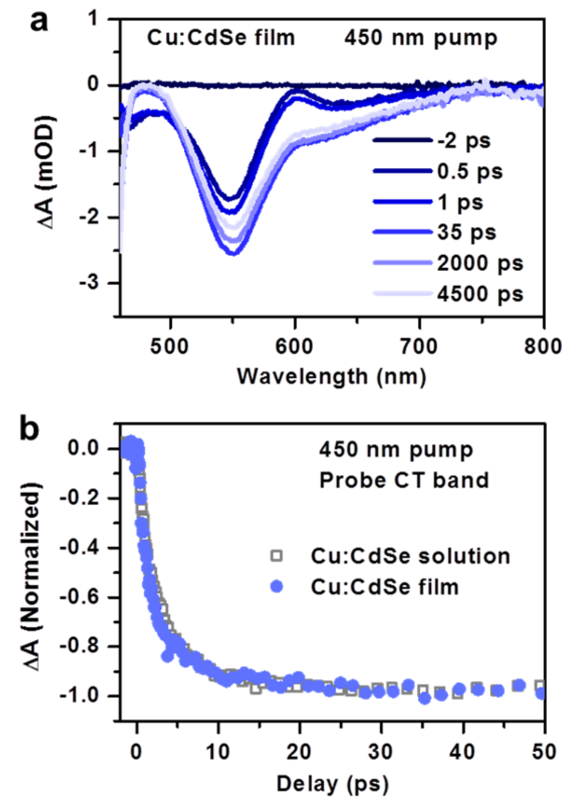

Fig. R3. (a) TA spectra of a densely-packed Cu:CdSe QD film at indicated time delays following the excitation by a 450 nm pulse. (b) TA kinetics probed at the CT band bleaches of Cu:CdSe QD solution (gray open squares) and film (blue solid circles).

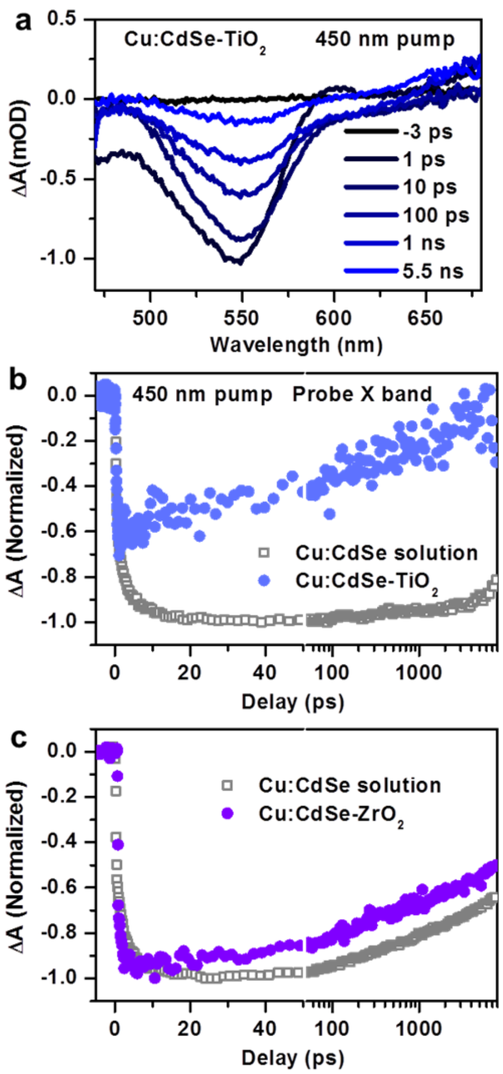

Fig. R4. (a) TA spectra of Cu:CdSe QD-sensitized mesoporous TiO_2 films at indicated time delays following the excitation by a 450 nm pulse. (b) TA kinetics probed at the X band bleaches of Cu:CdSe QD solution (gray open squares) and sensitized mesoporous TiO_2 films (blue solid circles). (c) TA kinetics probed at the X band bleaches of Cu:CdSe QD solution (gray open squares) and sensitized mesoporous ZrO_2 films (violet solid circles). Note that the QDs used for the ZrO_2 films were made from a different batch of sample.

For QD-sensitized mesoporous TiO_2 films, the electron kinetics probed at the X band (the CT band has a much lower signal to noise ratio due to scattering of the films) differs significantly with that of QDs in solution (Fig. R4a,b). First, when compared with QDs in solution, the X band bleach of QDs on TiO_2 only shows a sub-ps formation component whereas the slower formation component corresponding to slow hot electron relaxation disappears; Secondly, the X band bleach recovers much faster than QDs in solution. These two observations likely reflect the transfer of hot electrons and band edge electrons, respectively, from QDs to TiO_2. In order to

confirm that they are not caused by, *e.g.*, additional trapping process introduced when QDs are deposited onto oxide films, we also measure QD-sensitized mesoporous ZrO_2 films as a control sample. In this case, the formation kinetics of the X band bleach is similar for QDs on ZrO_2 and QDs in solution and the bleach recovery is only slightly accelerated (Fig. R4c). Thus, deposition onto oxide films only causes slight electron trapping for QDs and the observations in Fig. R4b might be consistent with the transfer of both hot and band edge electrons from QDs to TiO_2. However, we would like to note that although the comparison between QDs on TiO_2 and ZrO_2 is often used to determine ET from QDs to TiO_2, because of the lack of a direct observation of electrons injected into TiO_2 conduction band, we do not intend to make any conclusions about hot electron extraction here. Nonetheless, the data shown in Fig. R3 and Fig. R4b should already have proved that hot electron relaxation dynamics is negligibly affected when the QDs are incorporated into solid state.

Comment 3.

I suggest the authors can compare the hot electron relaxation dynamics and related lifetimes in the different materials systems and give some comments about hot-electron extraction for potential applications in solar cells.

Response: We thank the reviewer for this constructive suggestion. Indeed, such a comparison should give the readers a better perspective on the potential applications of hot electrons in different materials.

As we mentioned already in our response to your Comment 2, in terms of hot electron harvesting, QDs are not suitable for heterojunction solar cells, as in this case the hot electrons need to travel through the film to reach the heterojunction interface, which is very challenging considering the relatively low carrier mobility in QD solids as compared to bulk semiconductors. In contrast, although our comparison in the paper has shown that various types of lead halide perovskites have faster hot carrier energy loss rates than our Cu-doped QDs, they should show promise for hot electron extraction in heterojunction solar cells. As demonstrated by Huang and coworkers (*Science* **2017**, *356*, 59-62), quasi-ballistic transport of hot carriers in $MAPbI_3$ resulted in a transport distance of up to 230 nm that could overcome grain boundaries.

In the case of QD-sensitized solar cells, our Cu-doped CdSe QDs should perform better because of their longer-lived hot electrons that should allow for more efficient extraction by the adjacent oxide films. Moreover, CdSe QDs should have better stability than lead halide perovskites, especially when they are immersed in liquid electrolytes in sensitized solar cells. For the same reasons, Cu-doped QDs are more suited than perovskites if they were to be applied in photocatalysis utilizing hot electrons.

Revision: Because all the comments above are related to the potential applications of hot electrons in devices, we add a new section termed "Implication for devices" to the manuscript to simultaneously include our responses above:

"**Implication for devices.** While we have observed a hot electron lifetime of ~8.6 ps for Cu:CdSe QDs in solution, for potential applications such as solar cells QDs are

often in solid state. As such, it is important to examine whether incorporation into solid state films could introduce additional relaxation pathways affecting the hot electron lifetime. To this end, we also made measurements for QDs in densely-packed films and QDs adsorbed onto mesoporous metal oxide films; see Supplementary Figs. 9 and 10, respectively, for the data and Methods for the fabrication of these samples. In both cases, if no additional charge transfer pathways are introduced, the hot electron relaxation kinetics remains largely unaffected in the solid state, suggesting that the long-lived electrons should still be available when Cu:CdSe QDs are incorporated into devices.

Densely-packed films correspond to the situation of heterojunction solar cells using QD solids. In this case, the hot electrons need to travel through the film to reach the heterojunction interface for hot electron extraction, which is very challenging considering the relatively low carrier mobility in QD solids as compared to bulk semiconductors.[45] In this sense, lead halide perovskites should perform better, despite that our comparison above indicate they have faster hot carrier energy loss rates than our Cu:CdSe QDs, as Huang *et al.* has reported a quasi-ballistic transport distance of up to 230 nm for hot carriers in $MAPbI_3$ films.[6]

On the other hand, QDs adsorbed onto mesoporous metal oxide films are well suited for sensitized solar cells, where they are in direct contact with metal oxides and no carrier transport is required. Kamat *et al.* reported an electron transfer (ET) time constant of ~3.6 ps from CdSe QDs (diameter ~2.8 nm) to mesoporous SnO_2 films,[46] which should be fast enough to harvest hot electrons from Cu:CdSe QDs. If the surface of QDs were treated with short chain ligands to enable a strong electronic coupling between QDs and metal oxides, an even faster ET time and hence higher hot ET yield could be expected, as demonstrated by Zhu et al. for the case of PbSe QDs on TiO_2 single crystals.[47] Our preliminary measurements on Cu:CdSe QDs on mesoporous TiO_2 films indeed show some evidence for hot electron extraction (Supplementary Fig. 10). However, because of the lack of a direct observation of electrons injected into TiO_2 conduction band, we do not make any conclusive argument here and future efforts will be needed for convincing demonstration of hot electron extraction."

In the Supplementary Information, we add Figs. R3 and R4 as Supplementary Figs. 9 and 10, respectively.

Reviewer #3 (Remarks to the Author):

In this manuscript, Wu et al. report the hot electron relaxation dynamics in Cu doped CdSe quantum dots. Using the combination of ultrafast absorption and emission spectroscopy, the authors demonstrated that the presence of Cu in CdSe can significantly elongate hot electron cooling time, which can be attributed to the effective hole capturing by Cu, resulting weaker electron-hole coupling and enforcing photon bottleneck. In my opinion, this work is interesting and significant for two

reasons: 1) the fundamental understanding of photon bottleneck is critical to resolve the Shockley-Queisser limit, which is anticipated to be the key limiting factor to further improve solar energy conversion efficiencies; 2) the authors have carried out a systematic studies combining careful experiment and thorough data analysis. I recommend publication in this journal if the authors can address the minor concern listed below.

Response: We thank the reviewer very much for his/her kind comments.

1) The elongated hot electron lifetime has been reported before in Cu doped CdSe quantum dots as author stated in the manuscript (see line 71-73). Why much slower relaxation time was observed in this work than previous Cu doped CdSe systems? What are the key factors that lead to the difference of hole relaxation dynamics between Cu doped CdSe systems reported by different groups?

Response: We thank the reviewer for this insightful comment. Our data have clearly established that the very slow hot electron relaxation time in our QDs is enabled by femtosecond hole capturing by Cu dopants which outcompete the sub-ps electron-to-hole energy transfer. Thus, although not reported in refs. 35 and 36, the QDs used there might have a much slower hole capturing rate, thus allowing hot electron relaxation via energy transfer to the hole. The question is then why our Cu-doped QDs has a much faster hole capturing rate than those in refs .35 and 36. And we think the answer is the different synthetic methods resulting in different effective Cu concentrations in the QDs, because, in the simplest way, the hole transfer rate should imply scale with the number of available Cu dopant inside the QD. Our QDs has a Cu to Cd ratio of 1.8:100; the ratios were not reported in those studies but should be much lower. A spectroscopic signature for this ratio is the strength of the CT band absorption relative to that of the X band absorption. Based on the absorption spectra reported here and reported in those works, our sample has a much more prominent CT band absorption feature, confirming the high Cu ratio in our sample.

Revision: At the end of the section termed "Femtosecond hole capturing in Cu:CdSe QDs", we add the following contents to address the concern raised by the reviewer:
"Although not reported in refs. 35 and 36, we suspect the QDs used there might have a much slower hole capturing rate, thus allowing hot electron relaxation via energy transfer to the hole. This is likely due to the low Cu concentration in the QDs used there, because the hole transfer rate should roughly scale with the number of available Cu dopants inside the QD."

REVIEWERS' COMMENTS:

Reviewer #1 (Remarks to the Author):

The authors have addressed my comments sufficiently and I fully support the publication of this manuscript as is in Nature Communications. I look forward to seeing this work in print.

Reviewer #2 (Remarks to the Author):

The authors have addressed all my concerns. I think this manuscript should be published in Nature Communications.

Reviewer #3 (Remarks to the Author):

The authors have adequately addressed my concerns. This reviewer recommends publication in Nature Communication!

Reviewers’ comments in black; our responses in red.

Reviewers' comments:

Reviewer #1 (Remarks to the Author):

The authors have addressed my comments sufficiently and I fully support the publication of this manuscript as is in Nature Communications. I look forward to seeing this work in print.
Response: We thank the reviewer very much for supporting the publication of this manuscript in Nature Communications.

Reviewer #2 (Remarks to the Author):

The authors have addressed all my concerns. I think this manuscript should be published in Nature Communications.
Response: We thank the reviewer very much for agreeing with the publication of this paper in Nature Communications.

Reviewer #3 (Remarks to the Author):

The authors have adequately addressed my concerns. This reviewer recommends publication in Nature Communication!
Response: We thank the reviewer recommending publication of our work in Nature Communications.
