# Supplementary material for: Observation of a phonon bottleneck in copper-doped colloidal quantum dots
Source: Nat Commun. 2019 Oct 4;10:4532. doi: 10.1038/s41467-019-12558-y (PMC6778069; doi:10.1038/s41467-019-12558-y)
Supplement: Supplementary file 2 — Peer Review File [file 41467_2019_12558_MOESM2_ESM.pdf]

Supplementary Information

Observation of a phonon bottleneck in copper-doped colloidal quantum dots

Wang et al.

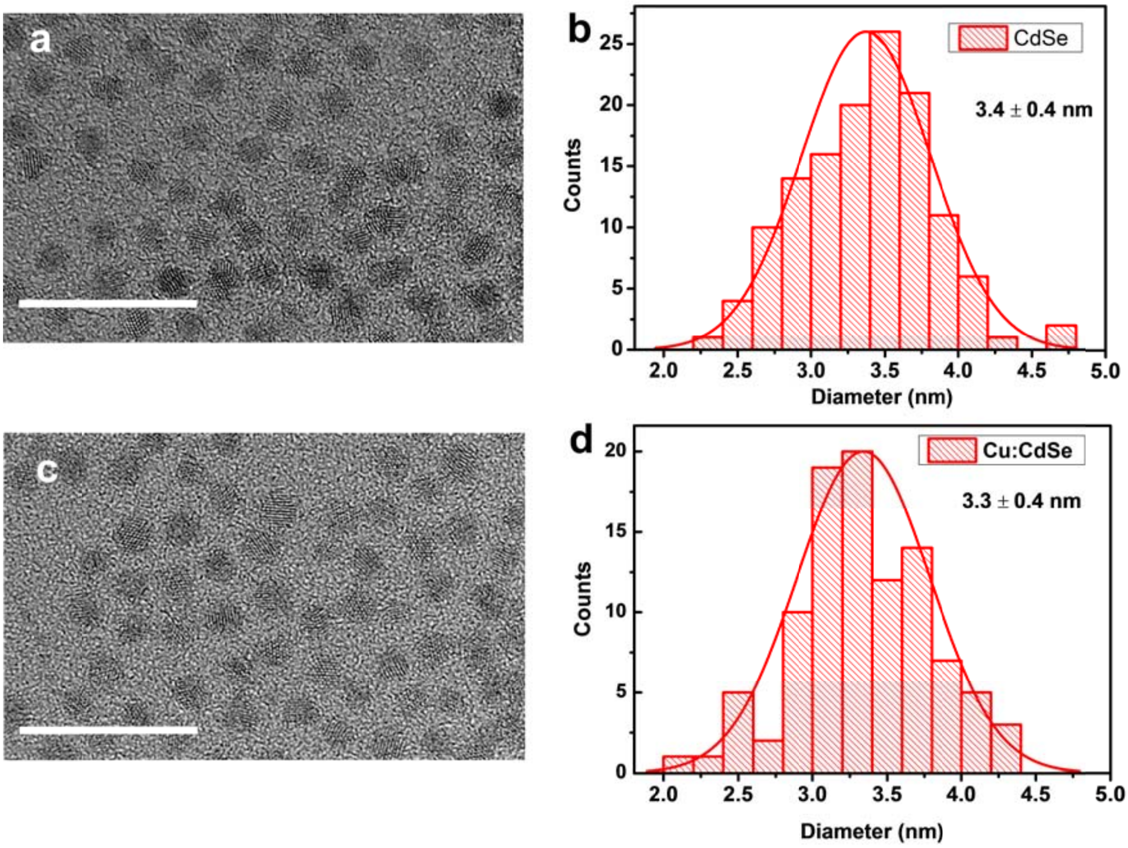

Supplementary Figure 1. (a,b) A representative TEM image of CdSe QDs (a) and the statistical histogram for their diameters (b). (c,d) A representative TEM **i**mage of Cu-doped CdSe (Cu:CdSe) QDs (c) and the statistical histogram for their diameters (d). The scale bars are 20 nm.

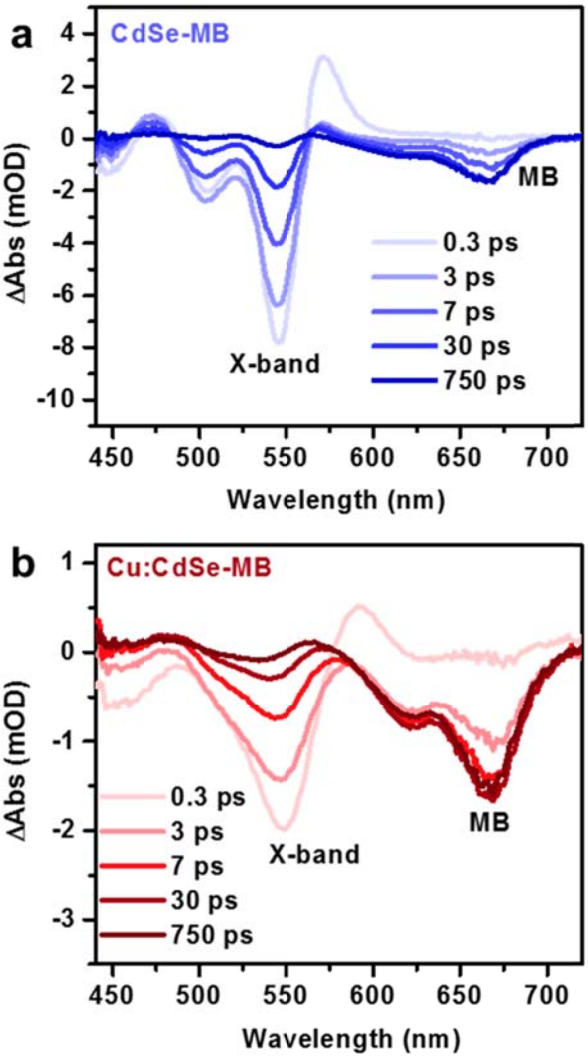

Supplementary Figure 2. TA spectra of (a)CdSe QD-methylene blue (MB) and (b) Cu:CdSe QD- MB complexes probed at indicated time delays following 400 nm excitation. At later time, both X-band and CT-band bleach features recover due to electron transfer from QDs to MB, resulting in TA spectra dominated by the ground state bleach feature of MB. These observations indicate that both X-band and CT-band bleaches are contributed by band edge electrons.

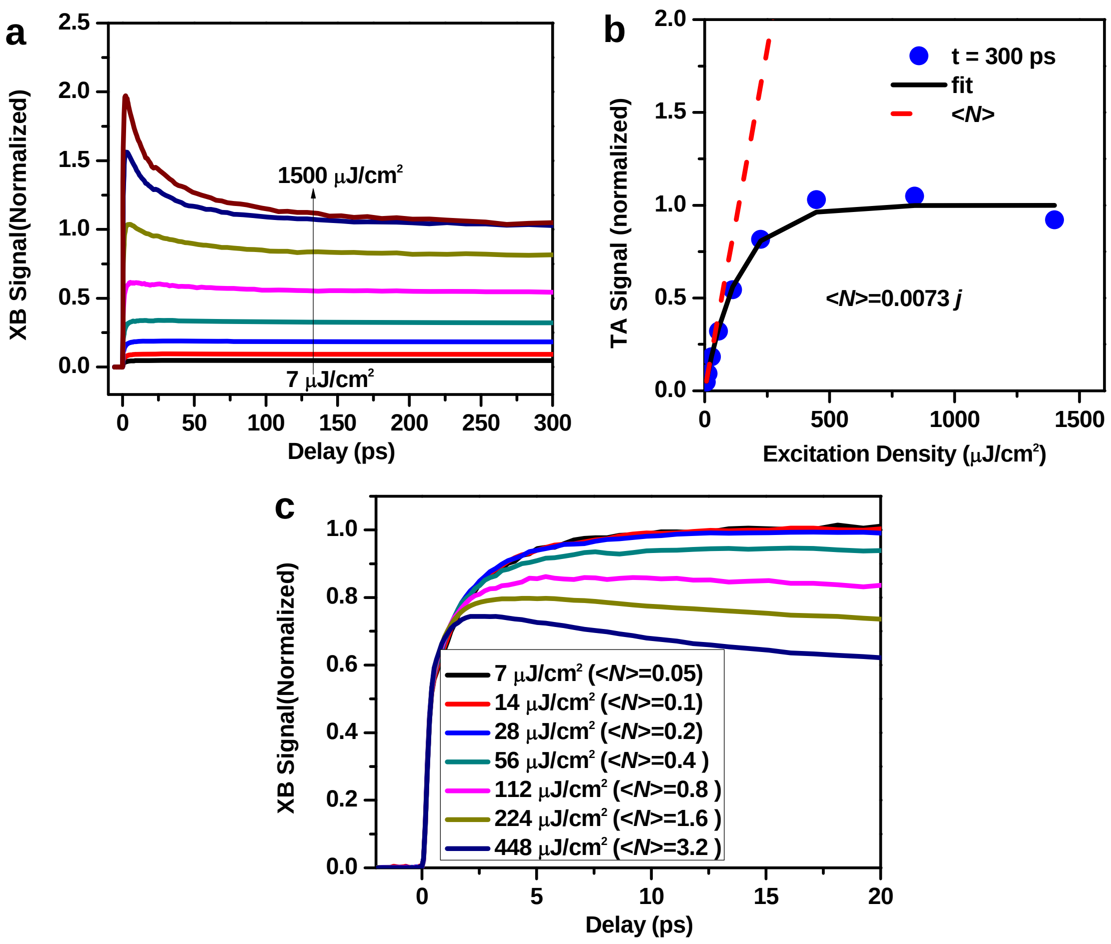

Supplementary Figure 3. (a) TA kinetics probed at the X-band bleach of Cu:CdSe QDs excited with a 400 pump pulse with various fluences from 7 μJ/cm^2 to 1500 μJ/cm^2. They are rescaled to set the saturated signal amplitude at 300 ps to 1. (b) Plot of the rescaled signal amplitude as a function of the excitation fluence (blue circles) and its fit to a Poisson statistics model (black solid line); see Supplementary Note 1. (c) TA kinetic traces under pump fluences from 7 μJ/cm^2 to 448 μJ/cm^2 (<*N*> from 0.05 to 3.2), appropriately rescaled to shown the effects of multi-exciton excitation on hot electron relaxation kinetics in the first 20 ps.

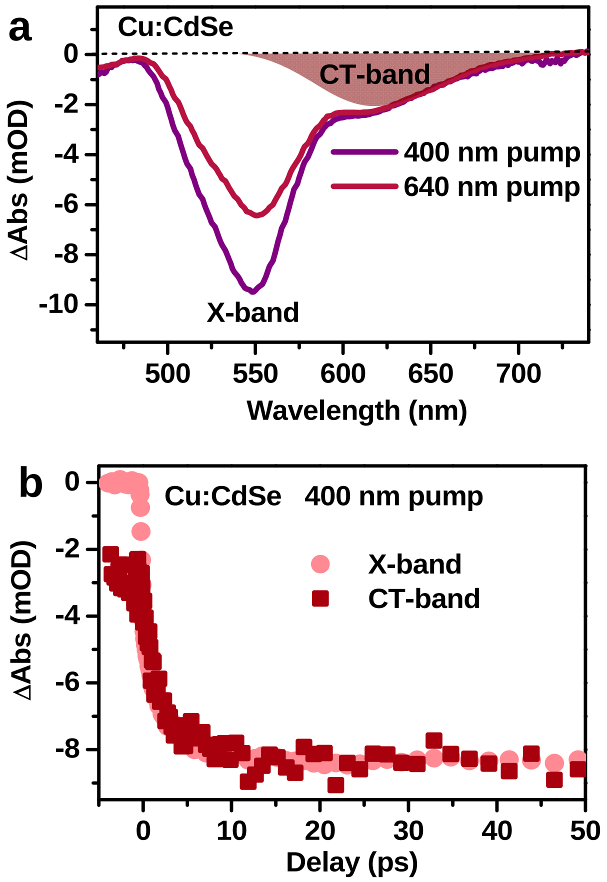

Supplementary Figure 4. (a) Comparison of TA spectra created by 400 nm (purple) and 640 nm (red) excitations at a time delay of 1 ns, normalized at the CT band. The red shaded Gaussian band is a fit to the CT-band. (b) Comparison of TA kinetics probed at X (light red circles) and CT (dark red squares) band bleaches under 400 nm excitation. The CT-band kinetics is scaled and downshifted to match the slowly-forming component of the X-band kinetics.

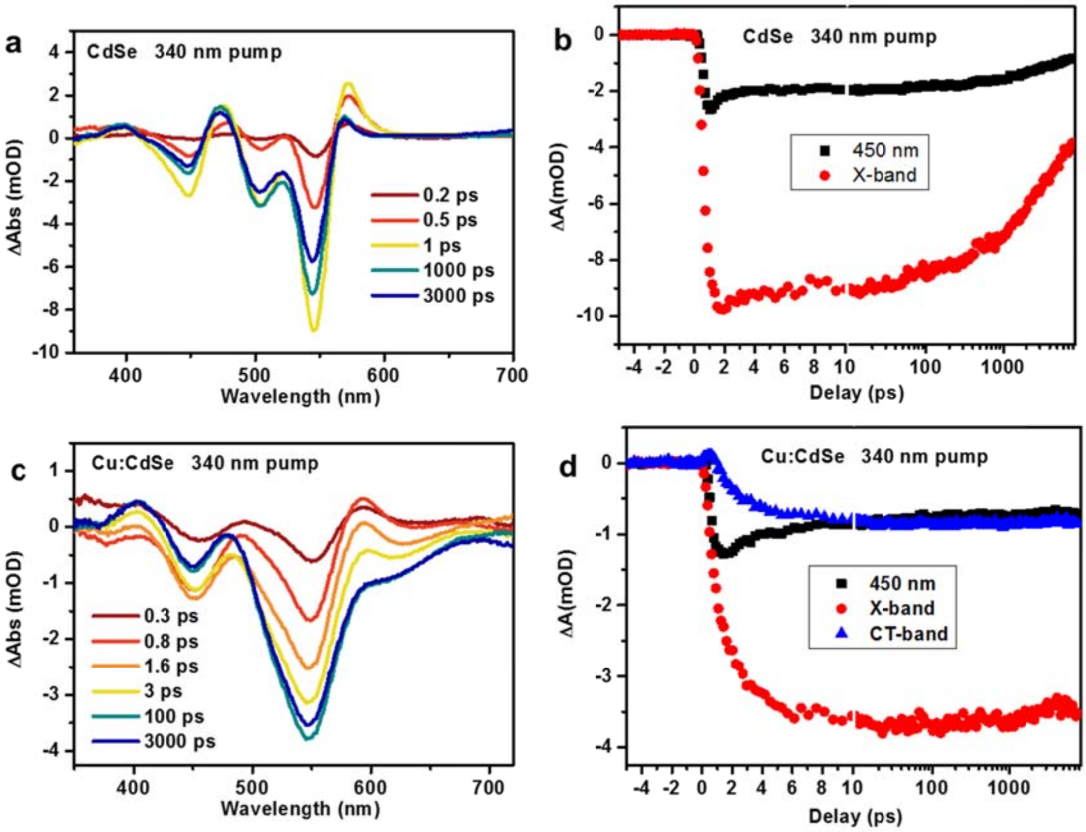

Supplementary Figure 5. (a) TA spectra of undoped CdSe QDs probed at indicated time delays following the excitation by a 340 nm pulse. (b) TA kinetics probed at the X-band bleach (red circles) and 450 nm feature (black squares). (c) TA spectra of Cu:CdSe QDs probed at indicated time delays following the excitation by a 340 nm pulse. (d) TA kinetics probed at the X-band bleach (red circles), the CT-band bleach (blue triangles) and 450 nm feature (black squares). In (b), the 450 nm feature of undoped QDs does not show obvious changes following slight decay within 2 ps. In contrast, in (d), the 450 nm feature of doped QDs shows slow decay within 100 ps that is complementary to the slow growth of the X-band and CT-band bleaches, indicative of long-lived hot electrons on the $1P_e$ level.

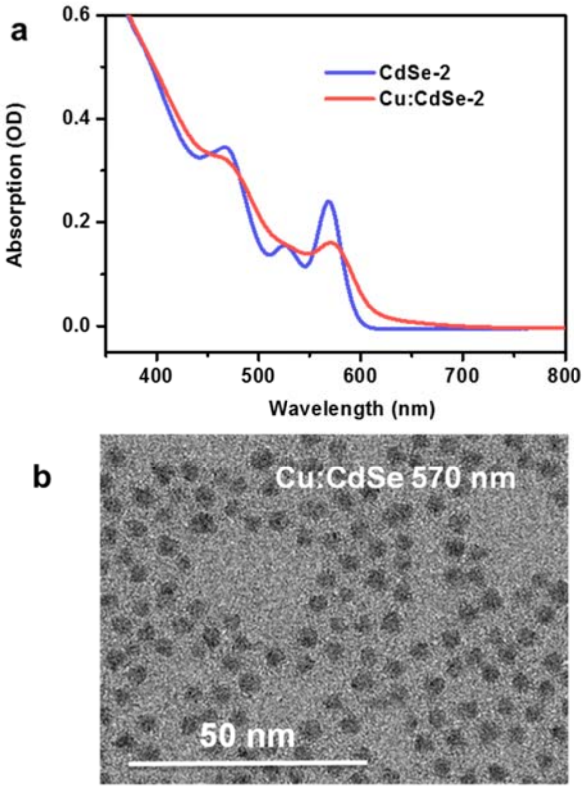

Supplementary Figure 6. (a) Absorption spectra of undoped (blue) and Cu-doped (red) CdSe QDs with their X-band feature at ~570 nm. These two samples are labeled as CdSe-2 and Cu:CdSe-2. (b) A TEM image of the Cu:CdSe-2 sample.

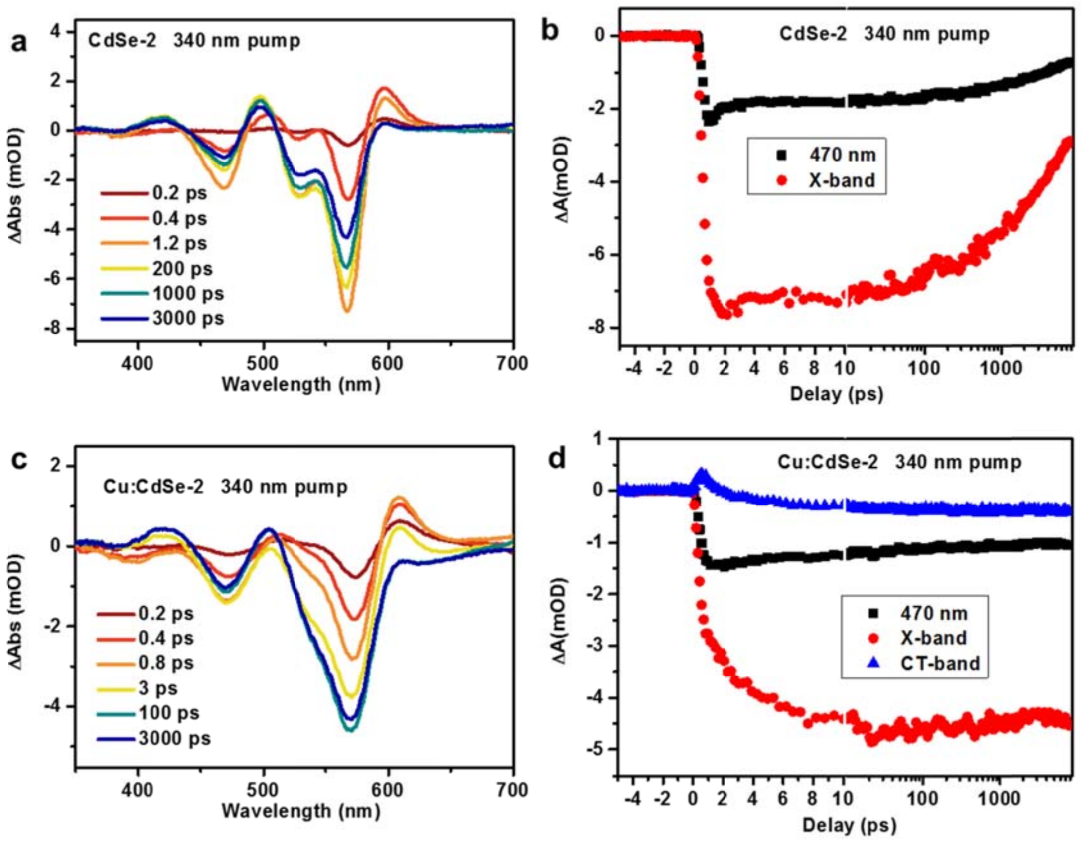

Supplementary Figure 7. (a) TA spectra of undoped CdSe-2QDs probed at indicated time delays following the excitation by a 340 nm pulse. (b) TA kinetics probed at the X-band bleach (red circles) and 470 nm feature (black squares). (c) TA spectra of Cu:CdSe-2QDs probed at indicated time delays following the excitation by a 340 nm pulse. (d) TA kinetics probed at the X-band bleach (red circles), the CT-band bleach (blue triangles) and 470 nm feature (black squares). In (b), the 470 nm feature of undoped QDs does not show obvious changes following slight decay within 2 ps. In contrast, in (d), the 470 nm feature of doped QDs shows slow decay within 100 ps that is complementary to the slow growth of the X-band and CT-band bleaches, indicative of long-lived hot electrons on the $1P_e$ level.

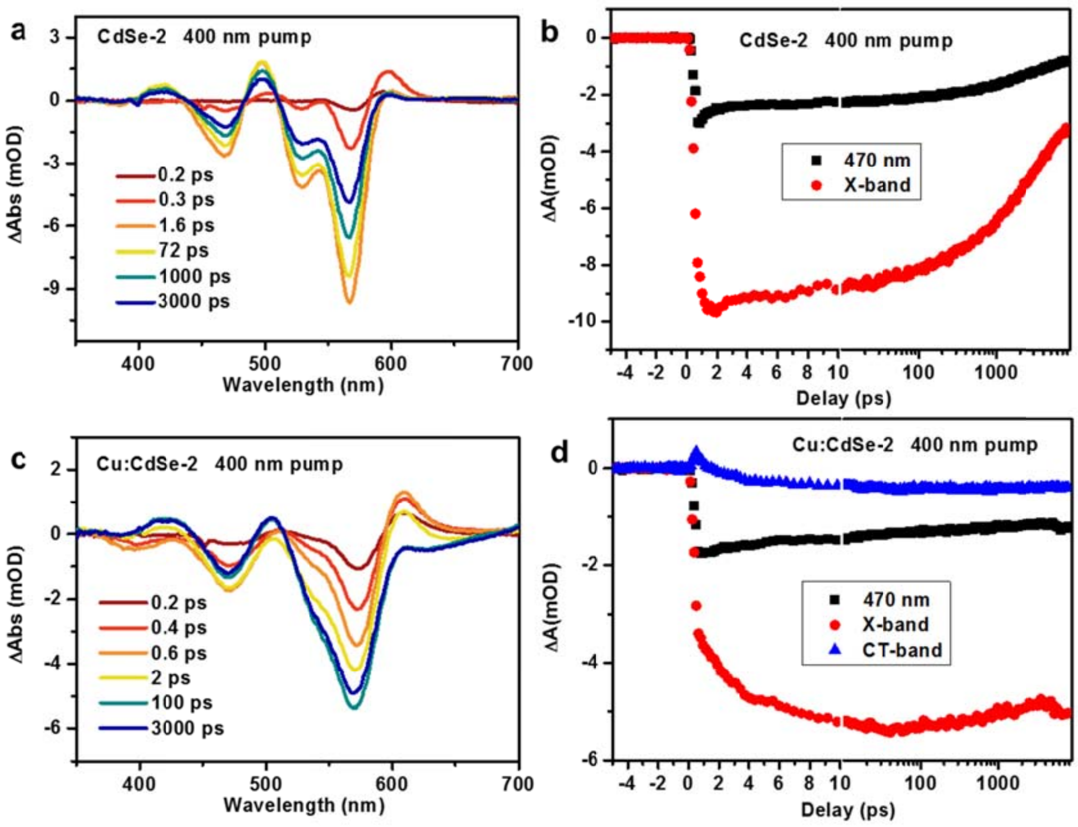

Supplementary Figure 8. (a) TA spectra of undoped CdSe-2QDs probed at indicated time delays following the excitation by a 400 nm pulse. (b) TA kinetics probed at the X-band bleach (red circles) and 470 nm feature (black squares). (c) TA spectra of Cu:CdSe-2QDs probed at indicated time delays following the excitation by a 400 nm pulse. (d) TA kinetics probed at the X-band bleach (red circles), the CT-band bleach (blue triangles) and 470 nm feature (black squares). In (b), the 470 nm feature of undoped QDs does not show obvious changes following slight decay within 2 ps. In contrast, in (d), the 470 nm feature of doped QDs shows slow decay within 100 ps that is complementary to the slow growth of the X-band and CT-band bleaches, indicative of long-lived hot electrons on the $1P_e$ level.

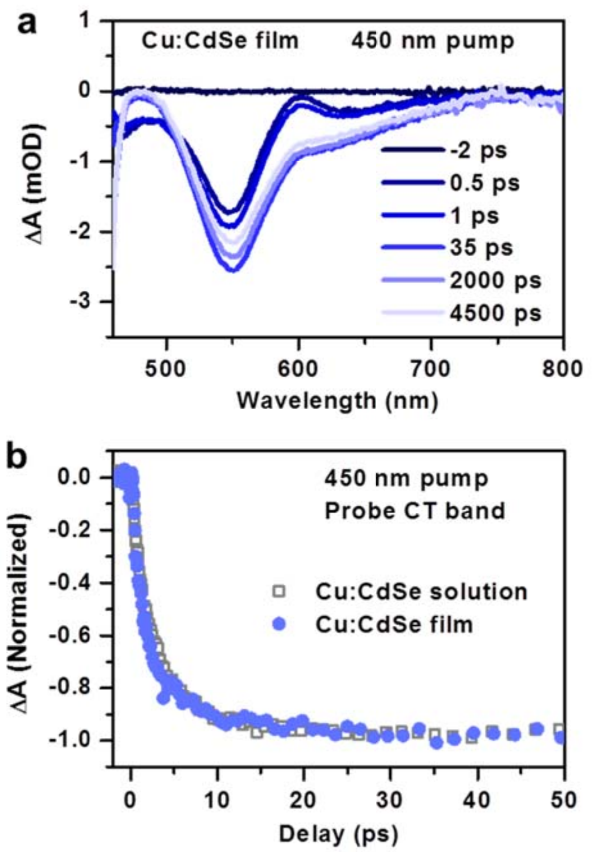

Supplementary Figure 9. (a) TA spectra of a densely-packed Cu:CdSe QD film at indicated time delays following the excitation by a 450 nm pulse. (b) TA kinetics probed at the CT band bleaches of Cu:CdSe QD solution (gray open squares) and film (blue solid circles). The hot electron relaxation kinetics (probed at the CT band) is almost the same for the solution and film samples, suggesting that the extra energy relaxation pathways introduced in the solid state (such as surface trap states) does not notably affect hot electron relaxation.

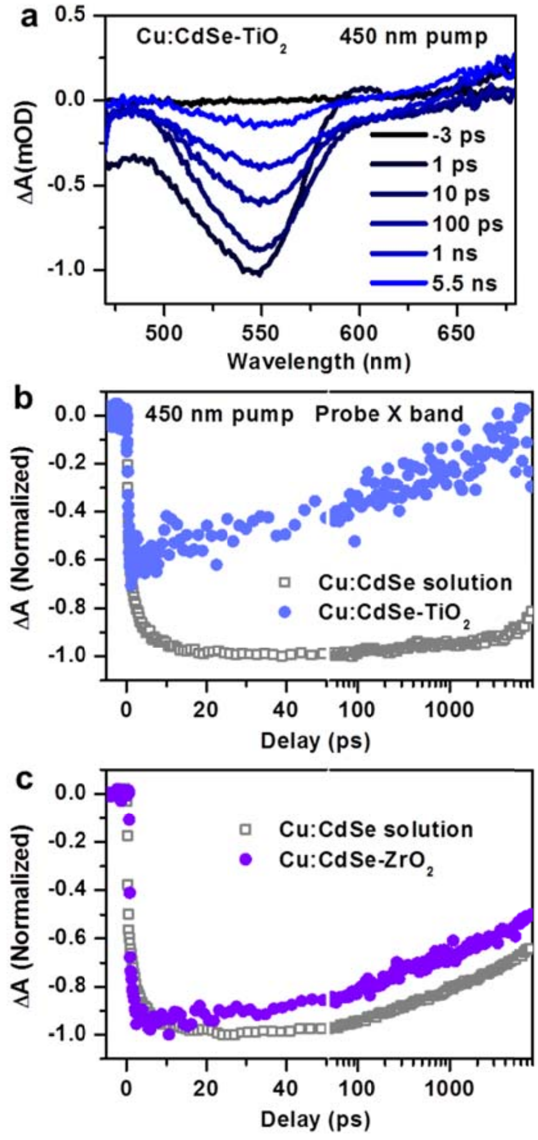

Supplementary Figure 10. (a) TA spectra of Cu:CdSe QD-sensitized mesoporous TiO_2 films at indicated time delays following the excitation by a 450 nm pulse. (b) TA kinetics probed at the X band bleaches of Cu:CdSe QD solution (gray open squares) and sensitized mesoporous TiO_2 films (blue solid circles). (c) TA kinetics probed at the X band bleaches of Cu:CdSe QD solution (gray open squares) and sensitized mesoporous ZrO_2 films (violet solid circles). Note that the QDs used for the ZrO_2 films were made from a different batch of sample.

Supplementary Note 1. Estimation of average exciton number per QD

The photon absorption events when using above band-gap excitation follow a Poisson distribution,[1] and hence, the TA signal at a delay time of 300 ps can be expressed as: $I \propto 1 - P(0) = 1 - e^{-\langle N \rangle}$, where $\langle N \rangle$ is the average number of photons absorbed per QD or the average exciton number per QD prior to Auger recombination. $\langle N \rangle$ is proportional to the pump laser fluence: $\langle N \rangle = Cj$. Therefore, the XB signal at 300 ps can be fitted with: $I \propto 1 - e^{-Cj}$, with C as the only fitting parameter. Using the fitted C value, the average exciton number $\langle N \rangle$ at each pump fluence can be calculated (blue solid line in the inset). For the 400 nm excitation data presented in Fig. 3c in the main text, the pump fluence was 7 μJ/cm^2, corresponding to $\langle N \rangle$ of 0.05. The 640 nm excitation data used a different pump fluence (~100 μJ/cm^2) due to much weaker absorption of the sample at this wavelength, but based on the signal amplitude in Fig. 3a, $\langle N \rangle$ should still be <0.1. This is consistent with the absence of multi-exciton Auger recombination processes in Fig. 3.

Supplementary Note 2. Kinetics fitting models

For undoped QDs, the X-band bleach kinetics under 400 nm excitation can be fitted to the following multi-exponential function accounting for both formation and decay of the signal:

$$S_{QD}(t) \propto \sum_{i=1}^{3} A_i e^{-k_i t} - e^{-k_f t} \qquad (1),$$

where k_f is the formation rate of the X-band bleach (*i.e.*, hot electron relaxation rate) and k_i (A_i) is the decay rate constant (and its relative amplitude) of the ith component accounting for the radiative and/or nonradiative decay of the $1S_e$ electrons. Note that eq. 1 is convoluted with the instrument response function of the TA set-up (approximated by a Gaussian with a FWHM of 90 fs) in the fitting; the same applies to all the equations below. The fitting parameters for the equations above are tabulated in Supplementary Table 1; note the rate constants have been converted into time constants in the table.

For the Cu-doped QDs, the X-band and CT-band bleach kinetics under 640 nm excitation are the same and they are fitted to the following expression:

$$S_{Cu:QD,640}(t) \propto \sum_{i=1}^{2} A_i e^{-k_i t} - e^{-k_f t} \qquad (2).$$

The X-band and CT-band bleach kinetics of the Cu-doped QDs under 400 nm excitation are different as the former contains contributions from undoped QDs in the sample. As a result, we first fit the CT-band bleach kinetics which is exclusively from doped QDs:

$$S_{Cu:QD,640}(t) \propto \sum_{i=1}^{2} A_i e^{-k_i t} - \sum_{i=1}^{2} B_i e^{-k_{f,i} t} \qquad (3).$$

In eq. 3, k_i and A_i are fixed using the parameters from eq. 2 as the band-edge $1S_e$ electron decay kinetics should be independent of excitation wavelength; $k_{f,i}$ and B_i are the decay rate constant and its relative amplitude, respectively, of the ith component of the hot electron relaxation process. The X-band bleach kinetics of the Cu-doped QDs under 400 nm excitation is then fitted to a linear combination of undoped QDs (eq. 1) and doped QDs (eq. 3). The fitting parameters for the equations above are tabulated in Supplementary Table 2.

Supplementary Table 1. Fitting parameters for undoped QDs

τ_f (ps)	τ_1 (ps)<br>A_1 (%)	τ_2 (ps)<br>A_1 (%)	τ_3 (ps)<br>A_3 (%)
0.25	81.9<br>10.2	2840<br>44.0	>>8000<br>45.8

Supplementary Table 2. Fitting parameters for doped QDs[a]

$\tau_{f,1}$ (ps)<br>B_1 (%)	$\tau_{f,2}$ (ps)<br>B_2 (%)	τ_1 (ps)<br>A_1 (%)	τ_2 (ps)<br>A_2 (%)
2.10<br>90.0	67.4<br>10.0	457<br>9.10	>>8000<br>90.9

a. CT-band, 400 nm excitation

Supplementary Note 3. Calculating hot carrier energy loss rates

Hot carrier energy loss rates (dE/dt) are calculated using: $dE/dt = \Delta E/\tau$, where ΔE is the excessive energy of the hot carriers above their band edges and τ is the hot carrier relaxation time constant under vanishingly weak excitation powers. ΔE can be estimated from the pump photon energies and the band structures of the investigated materials. For our Cu-doped and undoped CdSe QDs, hot electron excessive energy is estimated as: $\Delta E = (E_{pump} - E_{g,QD})m_e^*/(m_e^* + m_h^*)$, where E_{pump} is the pump photon energy, $E_{g,QD}$ is the gap of QDs, and m_e^* and m_h^* are the effective masses of electron (~0.13 free electron mass) and hole (~0.45 free electron mass), respectively, in CdSe. For $FAPbI_3$, $MAPbI_3$, $FAPbBr_3$, $MAPbBr_3$,

andCsPbBr$_3$ bulk films reported in ref 15 in the main text, the authors used a pump-pump-probe experiment where the second IR pump photon directly excited the band edge carriers in the conduction and valence bands, and hence $\Delta E = E_{pump,\ IR}$, where E_{pump} is the IR pump photon energy which is 0.6 eV in the paper. For FAPbBr$_3$, MAPbBr$_3$, andCsPbBr$_3$ NCs reported in ref 16 in the main text, ΔE can be estimated as: $\Delta E = (E_{pump} - E_{g,QD})/2$, where the factor 2 accounts for approximately symmetric conduction and valence band structures in lead halide perovskites. Using the equations above, we can calculate hot carrier energy loss rates for various materials under comparison. The results are tabulated in Supplementary Table 3 and plotted in Fig. 5.

Supplementary Table 3. Hot carrier energy loss rates for various materials.

	ΔE (eV)	τ (ps)	dE/dt (eV/ps)
FAPbI$_3$	0.6	0.22	2.73
MAPbI$_3$	0.6	0.32	1.88
FAPbBr$_3$	0.6	0.11	5.45
MAPbBr$_3$	0.6	0.18	3.33
CsPbBr$_3$	0.6	0.14	4.28
FAPbBr$_3$ NCs	0.61	0.21	2.90
MAPbBr$_3$ NCs	0.62	0.27	2.30
CsPbBr$_3$ NCs	0.56	0.39	1.44
CdSe QDs	0.65	0.25	2.60
Cu:CdSe QDs	0.65	8.6	0.075

Supplementary Note 4. QD-sensitized mesoporous films

For QD-sensitized mesoporous TiO$_2$ films, the electron kinetics probed at the X band (the CT band has a much lower signal to noise ratio due to scattering of the films) differs significantly with that of QDs in solution (Supplementary Fig. 10). First, when compared with QDs in solution, the X band bleach of QDs on TiO$_2$ only shows a sub-ps formation component whereas the slower formation component corresponding to slow hot electron relaxation

disappears; Secondly, the X band bleach recovers much faster than QDs in solution. These two observations likely reflect the transfer of hot electrons and band edge electrons, respectively, from QDs to TiO_2. In contrast, the formation kinetics of the X band bleach is similar for QDs on ZrO_2 and QDs in solution and the bleach recovery is only slightly accelerated. Thus, deposition onto oxide films only causes slight electron trapping for QDs and the observations for the TiO_2 sample might be consistent with the transfer of both hot and band edge electrons from QDs to TiO_2.

Supplementary References:

1 Klimov, V. I., Mikhailovsky, A. A., McBranch, D. W., Leatherdale, C. A. & Bawendi, M. G. Quantization of multiparticle Auger rates in semiconductor quantum dots. *Science* **287**, 1011-1013 (2000).
